# Phylogeography of Ryukyu insular cicadas: Extensive vicariance by island isolation vs accidental dispersal by super typhoon

**DOI:** 10.1371/journal.pone.0244342

**Published:** 2021-05-05

**Authors:** Soichi Osozawa, Kenichi Kanai, Haruo Fukuda, John Wakabayashi

**Affiliations:** 1 KawaOso Molecular Bio-Geology Institute, Sendai, Japan; 2 Institute of Geology and Paleontology, Faculty of Science, Tohoku University, Sendai, Japan; 3 Kagoshima Prefectural Amami High School, Amami, Japan; 4 Kagoshima Entomological Society, Kagoshima, Japan; 5 Department of Earth and Environmental Sciences, California State University, Fresno, California, United States of America; National Cheng Kung University, TAIWAN

## Abstract

Cicadas tend to be affected by vicariance reflecting poor mobility of nymphs underground and weak flying ability of adults. However, modern collection records of invasive cicada, combined with records of typhoon tracks, and newly obtained phylogeographic data suggest long distance, relatively instantaneous, dispersal of some vicariantly speciated cicadas. We address the importance of this typhoon dispersal mechanism applied to representative species of east Asian endemic cicadas of *Cryptotympana*, *Mogannia*, *Euterpnosia* and *Meimuna*. We combine BEAST-dated phylogenic and haplotype network analyses, modern collection data of non-native cicadas available in reports of the Japanese insect associations, modern typhoon records by Japan Meteorological Agency, and our own Quaternary geological constriction data. In conclusion, although Ryukyu endemic cicadas were vicariantly speciated, endemic cicadas on some islands were accidentally dispersed long distances to another island possibly by typhoons, particularly those associated with super typhoons generated since 1.55 Ma.

## Introduction

In an earlier paper [1], we showed extensive vicariant speciation acted on *Platypleura* cicada to generate each endemic population on each island of the Ryukyu chain, as well as the main islands of Japan and Taiwan. With a similar objective we analyzed the four cicada groups of *Cryptotympana*, *Mogannia*, *Euterpnosia* and *Meimuna*, which include endemic species and expected to find evidence of vicariance.

**Fig 1:**
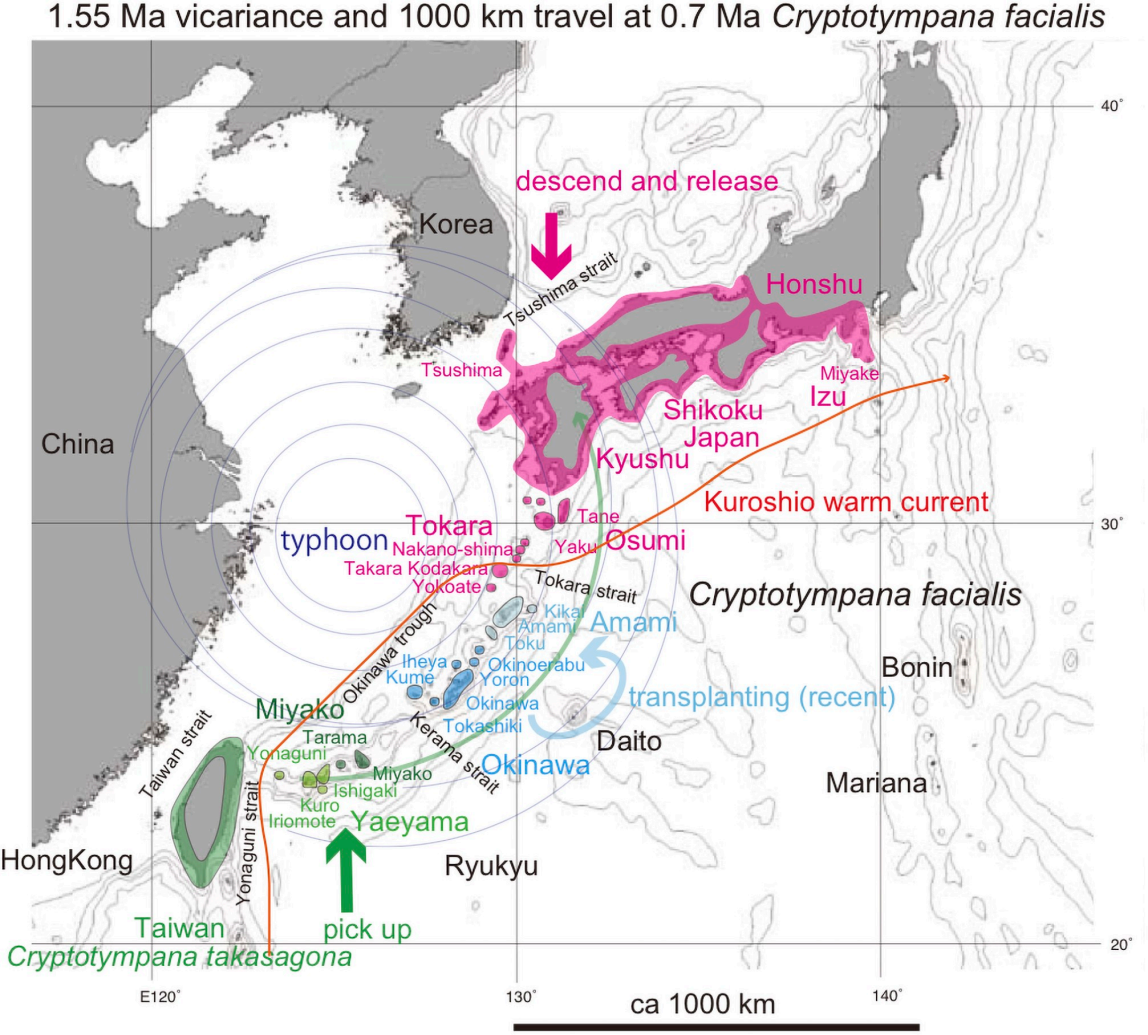
Distribution of *Cryptotympana facialis* in the Ryukyu and Japanese islands and *C*. *takasagona* in Taiwan. These species are lowland species as shown for the Japanese islands. Island populations are shown by color highlights. See Discussion; Green anticlockwise curved track: Super typhoon dispersal from Yaeyama (pale green) to Japan (red). The bold short arrows schematically (not to scale) show where the typhoon winds picked up cicada (green) and where the same cicada were released (red). Recent dispersal by human transport is also shown by the shorter counterclockwise path. The Kuroshio warm current is shown by the thin orange path with the arrow indicating northward to northeastward movement. Base map from Vector Map (VMap) Level 0, National Geospatial-Intelligence Agency.

Contrary to expectation, *Platypleura kaempferi* is widespread in the Japanese islands, Korea and China, including the northern Tokara islands (see Fig 1), and genetically similar (with the same molecular sequence) [1], suggesting a relatively strong dispersal ability of this cicada, including the ability to cross seaways.

*Cryptotympana facialis* is known to have recently invaded the Amami islands, that originally lacked this cicada [2, 3], and *Mogannia minuta* on the Okinawa main island recently extended its habitat [4]. The former example interpreted to have resulted from artificial transplanting, probably from the Okinawa islands, whereas the cause of the latter factor was uncertain. From phylogeographic study, we may be able to detect the origin area of a cicada species using the extent of vicariant speciation. Recognition of the cicada source may give insight into the mechanism of dispersal, being it an artificial or natural mechanism.

Cicadas are commonly absent from oceanic islands, but exceptions include *Euterpnosia chibensis daitoensis* on the Daito islands, *Meimuna boniensis* on the Bonin islands, and *Meimuna opalifera* on the Izu islands (island localities in Fig 1). A phylogeographic study for such cicadas may help ascertain the origin, date of dispersal, and dispersal mechanism.

We have shown that range fragmentation acted severely on these island cicada groups, but super typhoons generated since ca.1.55 Ma [5] appeared to have played an important role in long distance dispersal to oceanic islands. We also found evidence of intra continental island dispersal such as for *Cryptotympana facialis*. Dispersal of species by typhoon has not been adequately addressed as a dispersal mechanism to date. In this paper we consider multiple data threads, including recent typhoon track records, meteorology and physics, cicada ecology and collection records, transplantation records, and also formative (geologic) history of islands terraces.

## Materials

### Taxon sampling and the analytical aims

We collected four cicada groups from the Ryukyu islands, as well as from the Japan islands, Taiwan, and Chinese mainland (Table 1). See Japanese specimen photos in [6], and Taiwan specimen photos in [7].

**Table 1:** Cicada species collected and analyzed in this paper.

isolate	country	species	accession no. COI	accession no. 18SrRNA	collection date	collected by
kum25	Japan:Ryukyu, Amami-Oshima, Kasari	*Cryptotympana facialis* (Walker, 1858)	LC466803	LC466820	7/5/2018	Kenichi Kanai
kum26	Japan:Ryukyu, Amami-Oshima, Naze	*Cryptotympana facialis* (Walker, 1858)	ditto	ditto	7/6/2018	Kenichi Kanai
kum10	Japan:Ryukyu,Tokuno-shima, Amagi	*Cryptotympana facialis* (Walker, 1858)	ditto	ditto	7/22/2016	Taku Hotta
kum28A	Japan:Ryukyu,Tokuno-shima, Amagi	*Cryptotympana facialis* (Walker, 1858)	ditto	ditto	7/16/2018	Haruo Fukuda
kum28B	Japan:Ryukyu,Tokuno-shima, Amagi	*Cryptotympana facialis* (Walker, 1858)	ditto	ditto	7/16/2018	Haruo Fukuda
kum2	Japan:Ryukyu,Yoron-to	*Cryptotympana facialis* (Walker, 1858)	ditto	ditto	6/26/2013	Soichi Osozawa
kum30	Japan:Ryukyu,Okinawa-jima, Kunigami	*Cryptotympana facialis* (Walker, 1858)	ditto	ditto	7/5/2018	Kyoji Osozawa
kum31	Japan:Ryukyu,Okinawa-jima, Nago	*Cryptotympana facialis* (Walker, 1858)	ditto	ditto	7/10/2018	Yasushi Watanabe
kum20	Japan:Ryukyu,Okinawa-jima, Yaese	*Cryptotympana facialis* (Walker, 1858)	ditto	ditto	7/9/2017	Yasushi Watanabe
kum27A	Japan:Ryukyu, Amami-Oshima, Koniya	*Cryptotympana facialis* (Walker, 1858)	LC466804	LC466821	7/6/2018	Kenichi Kanai
kum27B	Japan:Ryukyu, Amami-Oshima, Koniya	*Cryptotympana facialis* (Walker, 1858)	ditto	ditto	7/6/2018	Kenichi Kanai
kum4	Japan:Ryukyu, Kume-jima	*Cryptotympana facialis* (Walker, 1858)	LC466805	LC466822	7/5/2014	Fumiyasu Sato
kum29A	Japan:Ryukyu, Okinawa-jima, Minna-jima	*Cryptotympana facialis* (Walker, 1858)	LC466806	LC466823	6/28/2018	Satoru Nitta
kum15	Japan:Ryukyu, Miyako-jima	*Cryptotympana facialis* (Walker, 1858)	LC466807	LC466824	7/4/2011	Soichi Osozawa
kum9	Japan:Ryukyu, Tarama-jima	*Cryptotympana facialis* (Walker, 1858)	LC466808	LC466825	6/14/2016	Soichi Osozawa
kum19	Japan:Ryukyu, Ishigaki-jima, Kabira	*Cryptotympana facialis* (Walker, 1858)	LC466809	LC466826	6/26/2017	Hiroshi Irino
kum32A	Japan:Ryukyu, Ishigaki-jima, Ishigaki	*Cryptotympana facialis* (Walker, 1858)	ditto	ditto	2018.7.18 19	Soichi Osozawa
kum32B	zJapan:Ryukyu, Ishigaki-jima, Ishigaki	*Cryptotympana facialis* (Walker, 1858)	ditto	ditto	2018.7.19	Soichi Osozawa
kum33	Japan:Ryukyu, Kuro-shima	*Cryptotympana facialis* (Walker, 1858)	LC466810	LC466827	7/18/2018	Soichi Osozawa
kum36A	Japan:Ryukyu, Iriomote-jima	*Cryptotympana facialis* (Walker, 1858)	LC466811	LC466828	9/7/2018	Hiroshi Irino
kum37	Japan:Ryukyu, Iriomote-jima	*Cryptotympana facialis* (Walker, 1858)	ditto	ditto	9/10/2018	Takafumi Nagai
kum36B	Japan:Ryukyu, Iriomote-jima	*Cryptotympana facialis* (Walker, 1858)	ditto	ditto	9/7/2018	Hiroshi Irino
kum18	Japan:Ryukyu, Yonaguni-jima,	*Cryptotympana facialis* (Walker, 1858)	LC466812	LC466829	7/10/2019	Minoru Saijo
kum21	Japan: Honshu,Itami	*Cryptotympana facialis* (Walker, 1858)	LC466813	LC466830	7/21/2018	Soichi Osozawa
kum14	Japan: Kyushu, Kagoshima	*Cryptotympana facialis* (Walker, 1858)	ditto	ditto	7/29/2016	Haruo Fukuda
kum23A	Japan: Osumi, Tanega-shima	*Cryptotympana facialis* (Walker, 1858)	ditto	ditto	7/27/2018	Yoshiyuki Ogata
kum23AB	Japan: Osumi, Tanega-shima	*Cryptotympana facialis* (Walker, 1858)	ditto	ditto	7/27/2018	Yoshiyuki Ogata
kum24A	Japan: Osumi, Yaku-shima	*Cryptotympana facialis* (Walker, 1858)	ditto	ditto	7/20/2018	Kyohei Nakamura
kum24B	Japan: Osumi, Yaku-shima	*Cryptotympana facialis* (Walker, 1858)	ditto	ditto	7/20/2018	Kyohei Nakamura
kum24C	Japan: Osumi, Yaku-shima	*Cryptotympana facialis* (Walker, 1858)	ditto	ditto	7/20/2018	Kyohei Nakamura
kum8	Japan: Tokara, Nakano-shima	*Cryptotympana facialis* (Walker, 1858)	ditto	ditto	8/29/2010	Soichi Osozawa
kum13	Japan: Tokara, Takara-jima	*Cryptotympana facialis* (Walker, 1858)	ditto	ditto	7/17/2016	Taiji Moriyama
kum24A	Japan: Tokara, Kodakara-jima	*Cryptotympana facialis* (Walker, 1858)	ditto	ditto	8/27/2017	Hideyuki Iwashita
kum24B	Japan: Tokara, Kodakara-jima	*Cryptotympana facialis* (Walker, 1858)	ditto	ditto	8/27/2017	Hideyuki Iwashita
kum34	Taiwan:Wulai	*Cryptotympana holsti* Distant, 1904	LC466814	LC466831	7/4/2018	Soichi Osozawa
kum1	China: HongKong	*Cryptotympana mandarina* Distant, 1891	LC466815	LC466832	5/29/2013	Soichi Osozawa
kum12	Taiwan:Taipei Zoo	*Cryptotympana takasagona* Kato, 1925	LC466816	LC466833	8/28/2016	Soichi Osozawa
kum3	South Korea:Busan	*Cryptotympana atrata* (Fabricius, 1775)	LC466817	LC466834	7/12/2013	Soichi Osozawa
kum5	China: Zhejiang	*Cryptotympana atrata* (Fabricius, 1775)	LC466818	LC466835	8/13/2013	Akira Mishima
N-ec1	Japan:Ryukyu,Minami Daito-jima	*Euterpnosia chibensis daitoensis* Matsumura, 1917	LC508809	LC508884	4/5/2012	Ryosuke Sadaki
S-ec2	Taiwan:Tailuko	*Euterpnosia olivacea* Kato, 1927	LC508810	LC508885	2010,8.8	Soichi Osozawa
S-ec3	Japan:Ryukyu,Yonaguni-jima	*Euterpnosia iwasakii* (Matsumura, 1913)	LC508811	LC508886	6/29/2011	Soichi Osozawa
S-ec4a	Japan:Ryukyu,Ishigaki-jima	*Euterpnosia iwasakii* (Matsumura, 1913)	LC508812	LC508887	6/25/2011	Soichi Osozawa
N-ec5a	Japan:Ryukyu,Kume-jima	*Euterpnosia chibensis okinawana* Ishihara, 1968	LC508813	LC508888	6/23/2012	Soichi Osozawa
N-ec6a	Japan:Ryukyu,Okinawa-jima	*Euterpnosia chibensis okinawana* Ishihara, 1968	LC508814	LC508889	6/25/2010	Soichi Osozawa
N-ec6a-2	Japan:Ryukyu,Okinawa-jima	*Euterpnosia chibensis okinawana* Ishihara, 1968	LC508815	LC508890	6/25/2010	Soichi Osozawa
N-ec7a	Japan:Ryukyu,Tokuno-shima	*Euterpnosia chibensis* Matsumura, 1917	LC508816	LC508891	7/2/2012	Soichi Osozawa
N-ec7b	Japan:Ryukyu,Tokuno-shima	*Euterpnosia chibensis* Matsumura, 1917	LC508817	LC508892	7/2/2012	Soichi Osozawa
N-ec8a	Japan:Ryukyu,Amami Oshima	*Euterpnosia chibensis* Matsumura, 1917	LC508818	LC508893	6/28/2010	Soichi Osozawa
N-ec9	Japan:Honshu,Kochi	*Euterpnosia chibensis* Matsumura, 1917	LC508819	LC508894	8/1/2011	Soichi Osozawa
S-ec10	Taiwan:Hualien	*Euterpnosia viridifrons* Matsumura, 1917	LC508820	LC508895	6/4/2013	Soichi Osozawa
S-ec12a	Taiwan:Yanhmungshan	*Euterpnosia gina* Kato, 1931	LC508821	LC508896	6/7/2013	Soichi Osozawa
S-ec13	Japan:Ryukyu,Ishigaki-jima	*Euterpnosia iwasakii* (Matsumura, 1913)	LC508822	LC508897	6/15/2013	Soichi Osozawa
N-ec14-1	Japan:Ryukyu,Ihaya-jima	*Euterpnosia chibensis okinawana* Ishihara, 1968	LC508823	LC508898	6/21/2013	Soichi Osozawa
N-ec15	Japan:Ryukyu,Okinoerabu-jima	*Euterpnosia chibensis okinawana* Ishihara, 1968	LC508824	LC508899	6/28/2013	Soichi Osozawa
N-ec17-1	Japan:Ryukyu,Amamai Oshima	*Euterpnosia chibensis* Matsumura, 1917	LC508825	LC508900	7/9/2013	Soichi Osozawa
N-ec18a	Japan:Kyushu,Kagoshima	*Euterpnosia chibensis* Matsumura, 1917	LC508826	LC508901	8/3/2013	Teruhiko Fukuda et al
ec19	China:Anhui	*Euterpnosia* sp.	LC508827	LC508902	6/10/2013	Akira Mishima
min-mn2	Japan:Ryukyu,Yonaguni-jima	*Mogannia minuta* (Matsumura, 1907)	LC508828	LC508903	6/29/2011	Soichi Osozawa
min-mn3	Japan:Ryukyu,Ishigaki-jima	*Mogannia minuta* (Matsumura, 1907)	LC508829	LC508904	5/1/2010	Soichi Osozawa
min-mn4	Japan:Ryukyu,N Iriomote-jima	*Mogannia minuta* (Matsumura, 1907)	LC508830	LC508905	4/30/2010	Soichi Osozawa
min-mn5	Japan:Ryukyu,Miyako-jima	*Mogannia minuta* (Matsumura, 1907)	LC508831	LC508906	4/25/2011	Soichi Osozawa
min-mn6	Japan:Ryukyu, Okinawa-jima, Tsuken-jima	*Mogannia minuta* (Matsumura, 1907)	LC508832	LC508907	6/6/2012	Satoru Nitta
min-mn9a	Japan:Ryukyu, Okinawa-jima, Yagachi-jima	*Mogannia minuta* (Matsumura, 1907)	LC508833	LC508908	5/11/2014	Atsuko Nitta
min-mn10a	Japan:Ryukyu,Okinawa-jima,Tamagusuku	*Mogannia minuta* (Matsumura, 1907)	LC508834	LC508909	5/30/2014	Ysushi Watanabe
min-mn12a	Japan:Ryukyu,Kohama-jima	*Mogannia minuta* (Matsumura, 1907)	LC508835	LC508910	7/6/2014	Soichi Osozawa
min-mn14a	Japan:Ryukyu,S Iriomote-jima	*Mogannia minuta* (Matsumura, 1907)	LC508836	LC508911	6/18/2016	Soichi Osozawa
mn1B	Taiwan:Hualien	*Mogannia formosana* Matsumura, 1907	LC508837	LC508912	6/2/2013	Soichi Osozawa
heb-mn2B	Taiwan:Hualien	*Mogannia hebes* Walker, 1858	LC508838	LC508913	6/2/2013	Soichi Osozawa
heb-mn3B	Taiwan:Hualien	*Mogannia hebes* Walker, 1858	LC508839	LC508914	6/3/2013	Soichi Osozawa
heb-mn5B	Taiwan:Yangmingshan	*Mogannia hebes* Walker, 1858	LC508840	LC508915	6/7/2013	Soichi Osozawa
heb-mn6B	Taiwan:Yangmei	*Mogannia hebes* Walker, 1858	LC508841	LC508916	6/8/2013	Soichi Osozawa
heb-mn7aB	China:HongKong	*Mogannia hebes* Walker, 1858	LC508842	LC508917	5/30/2013	Akira Mishima
heb-mn13aB	China:Hubei	*Mogannia hebes* Walker, 1858	LC508843	LC508918	6/18/2014	Soichi Osozawa
tuk1	Japan:Kyushu,Tsushima	*Meimuna opalifera* (Walker, 1850)	LC508844	LC508919	7/18/2013	Soichi Osozawa
tuk2	Japan:Honshu,Okino-shima	*Meimuna opalifera* (Walker, 1850)	LC508845	LC508920	8/28/2013	Soichi Osozawa
tuk3a	Japan:Izu,Hachijo-jima	*Meimuna opalifera* (Walker, 1850)	LC508846	LC508921	7/16/2014	Soichi Osozawa
tuk4	Japan:Tokara,Nakano-shima	*Meimuna opalifera* (Walker, 1850)	LC508847	LC508922	8/31/2010	Soichi Osozawa
tuk5	Japan:Honshu,Sendai	*Meimuna opalifera* (Walker, 1850)	LC508848	LC508923	9/6/2014	Soichi Osozawa
tuk6	China:Zhejiang	*Meimuna opalifera* (Walker, 1850)	LC508849	LC508924	8/13/2014	Soichi Osozawa
tuk10	Japan:Kyushu,Yaku-shima	*Meimuna opalifera* (Walker, 1850)	LC508850	LC508925	10/11/2017	Haruo Fukuda
tuk12	Taiwan:Taipei	*Meimuna opalifera* (Walker, 1850)	LC508851	LC508926	11/6/2017	Soichi Osozawa
kur1	Japan:Ryukyu,Amami Oshima	*Meimuna kuroiwae* (Matsumura, 1917)	LC508857	LC508931	9/9/2010	Soichi Osozawa
kur2	Japan:Tokara,Takara-jima	*Meimuna kuroiwae* (Matsumura, 1917)	LC508858	LC508932	9/3/2010	Soichi Osozawa
kur3a	Japan:Tokara,Nakano-shima	*Meimuna kuroiwae* (Matsumura, 1917)	LC508859	LC508933	8/29/2010	Soichi Osozawa
kur4a	Japan:Ryukyu,Kume-jima	*Meimuna kuroiwae* (Matsumura, 1917)	LC508860	LC508934	8/15/2014	Soichi Osozawa
kur5	Japan:Ryukyu,Okinawa-jima	*Meimuna kuroiwae* (Matsumura, 1917)	LC508861	LC508935	9/17/2015	Soichi Osozawa
kur6	Japan:Ryukyu,Okinawa-jima	*Meimuna kuroiwae* (Matsumura, 1917)	LC508862	LC508936	9/17/2015	Soichi Osozawa
kur7a	Japan:Ryukyu,Iheya-jima	*Meimuna kuroiwae* (Matsumura, 1917)	LC508863	LC508937	9/18/2015	Soichi Osozawa
kur8	Japan:Kyushu,Kagoshima,Cape Sata	*Meimuna kuroiwae* (Matsumura, 1917)	LC508864	LC508938	10/4/2017	Nobuharu Kumagai
kur9	Japan:Kyushu,Yaku-shima	*Meimuna kuroiwae* (Matsumura, 1917)	LC508865	LC508939	10/10/2017	Haruo Fukuda
kur10	Japan:Ryukyu,Kikai-jima	*Meimuna kuroiwae* (Matsumura, 1917)	LC508866	LC508940	11/10/2017	Nobuharu Kumagai

The present study did not involve endangered or protected species. We obtained permission of collection in the Taroko National Park, Taiwan, from the director (No. 0990012881; August 1 ~ 11, 2010), with a help of Bor-ming Jahn, and permission of collection in the Tokara islands, from the Toshima village headman, from August 29 ~ September 8, 2010. Collection in the Ryukyu islands was before the designation of National Park since 2016. No specific permission was required outside the national parks and private areas.

### *Cryptotympana* (Fig 1)

We collected *Cryptotympana takasagona* and *C*. *holsti* from Taiwan, *C*. *mandarina* from Hong Kong, and *C*. *atrata* from China and Korea [8]. Sequence data for *C*. *consanguinea* in Mindanao island of the Philippines [8] and *C*. *mandarinais* in Vietnam are available in GenBank/DDBJ.

*C*. *facialis* is endemic in the Japanese main islands as well as the Ryukyu islands, northern Izu islands, and Tsushima island (Fig 1). Its song and habitat are similar to *C*. *takasagona* in Taiwan [6]. A white band along the margin of abdominal segment III is a characteristic of the Yaeyama islands population and Tarama island specimens. This band is especially broad on Yonaguni island specimens, but completely absent on specimens from Okinawa and Miyako islands. This white band is narrow or absent for species inhabiting the Japan main islands, but somewhat broad for the Osumi and Tokara island populations. Emergence time tends to be early for the Yaeyama islands population situated to the south, and delayed for the more northerly Japanese population, but exceptionally delayed for the Iriomote island population (a part of the Yaeyama islands population). These morphological and ecological characters may be related to the vicariance on these islands, suggesting the potential utility of phylogenetic study.

*C*. *facialis* inhabits lowland regions including coastal area less than ca. 100 m altitude (red area in Fig 1 for the Japan main islands) such as the Ryukyu islet areas. *C*. *facialis* was originally absent from the Amami islands of Amami Oshima, Toku, and Kikai [2, 3] (Fig 1), possibly as a consequence of the geologic evolution of these islands (see Discussion). *C*. *facialis*, however, has been found on these islands since 1991, and has been proposed to have arrived as a result of transplantation from Okinawa [9]. The newly found Amami islands *C*. *facialis* lack the white abdominal band similar to the Okinawa island population [10]. We will show that *C*. *facialis* collected from the Amami islands is genetically common to those in the Okinawa islands.

### *Mogannia* (Fig 2)

**Fig 2:**
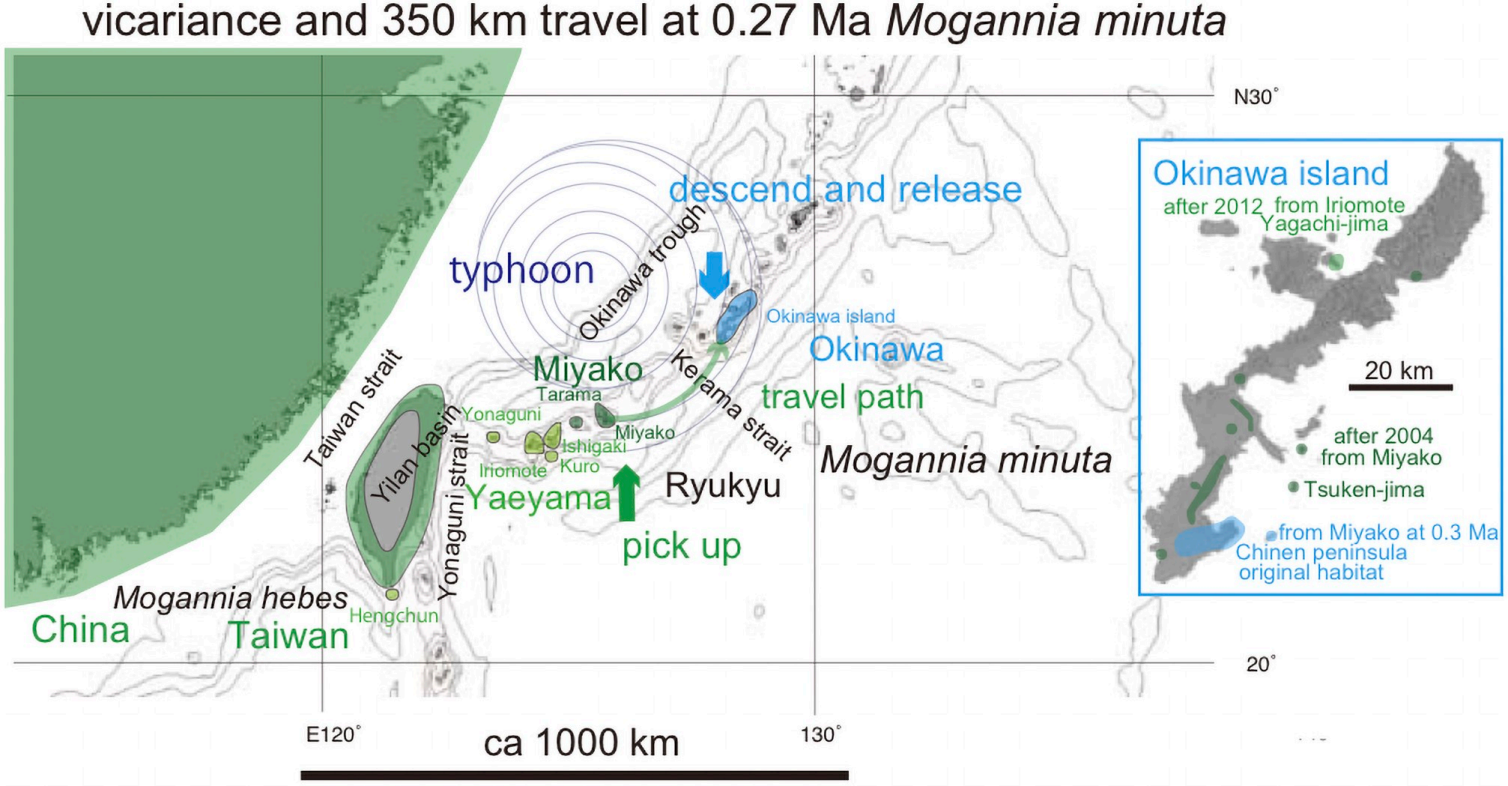
Distribution of *Mogannia*. Island populations are shown by color highlights. See Discussion; Green anticlockwise curved track: Typhoon dispersal at 0.27 Ma from Miyako (green) to Okinawa (blue). The bold short arrows schematically (not to scale) show where the typhoon winds picked up cicada (green) and where the same cicada were released (blue). Inset: Original habitat in the southern Okinawa and new habitats (blue). We clarified that only the Yagachi-jima population (green) has the same COI sequence with the southern Iriomote-jima, Yaeyama, and dispersed from there. Base map from Vector Map (VMap) Level 0, National Geospatial-Intelligence Agency.

*Mogannia minuta* is distributed on Okinawa island, and the Miyako and Yaeyama islands. Although this species was first reported and described from south Taiwan [11] and a specimen (11–15 June, 1937, Heng-chun, M. Chujo leg.) was examined [12], no record was known from Taiwan after World War II, so we could not include the Taiwan specimen in our analyses.

The emergence time of *M*. *minuta* is late March to late June with a peak of late April for the Yaeyama islands, end of April to May for the Miyako islands, and May for the Okinawa islands, although some cicadas continue to sing until June in Yagachi-jima (new habitat; Fig 2 inset) of the northern Okinawa islands. This variation of emergence may be related to the vicariance in these islands.

Habitats of *M*. *minuta* on Okinawa island were originally restricted to the southern part of the Chinen peninsula (Fig 2 inset). However, [4] reported 2004 reports of this species from north of the Chinen peninsula, up to 20 km from the former area of distribution. Internet records dating from 2012 were found for documenting the presence of cicada in northern Okinawa, including Yagachi-jima (Fig 2 inset). Our study is designed to test the mechanism of habitat expansion of this species. Note that nymphs of this species are usually underground for two years [12], so dispersed female(s) at a new location should have egged two years before the first records of emergence and singing. Also note that founding population size might have very small (minimum: a single gravid female), but *M*. *minuta* has successfully colonized such as in Yagachi-jima in 2012 and continued to survive there in 2020.

*Mogannia hebes* is known from Taiwan and southern China (Fig 2), and we collected specimens for comparison with *M*. *minuta*. Note that the Yilan basin and Lanyang valley (the on-land continuation of the Okinawa trough on Taiwan) constitute a physical biological barrier separating the northwestern (Chinese side) and southeastern (Ryukyu side) of Taiwan (Fig 2) [13].

### *Euterpnosia* (Fig 3)

**Fig 3:**
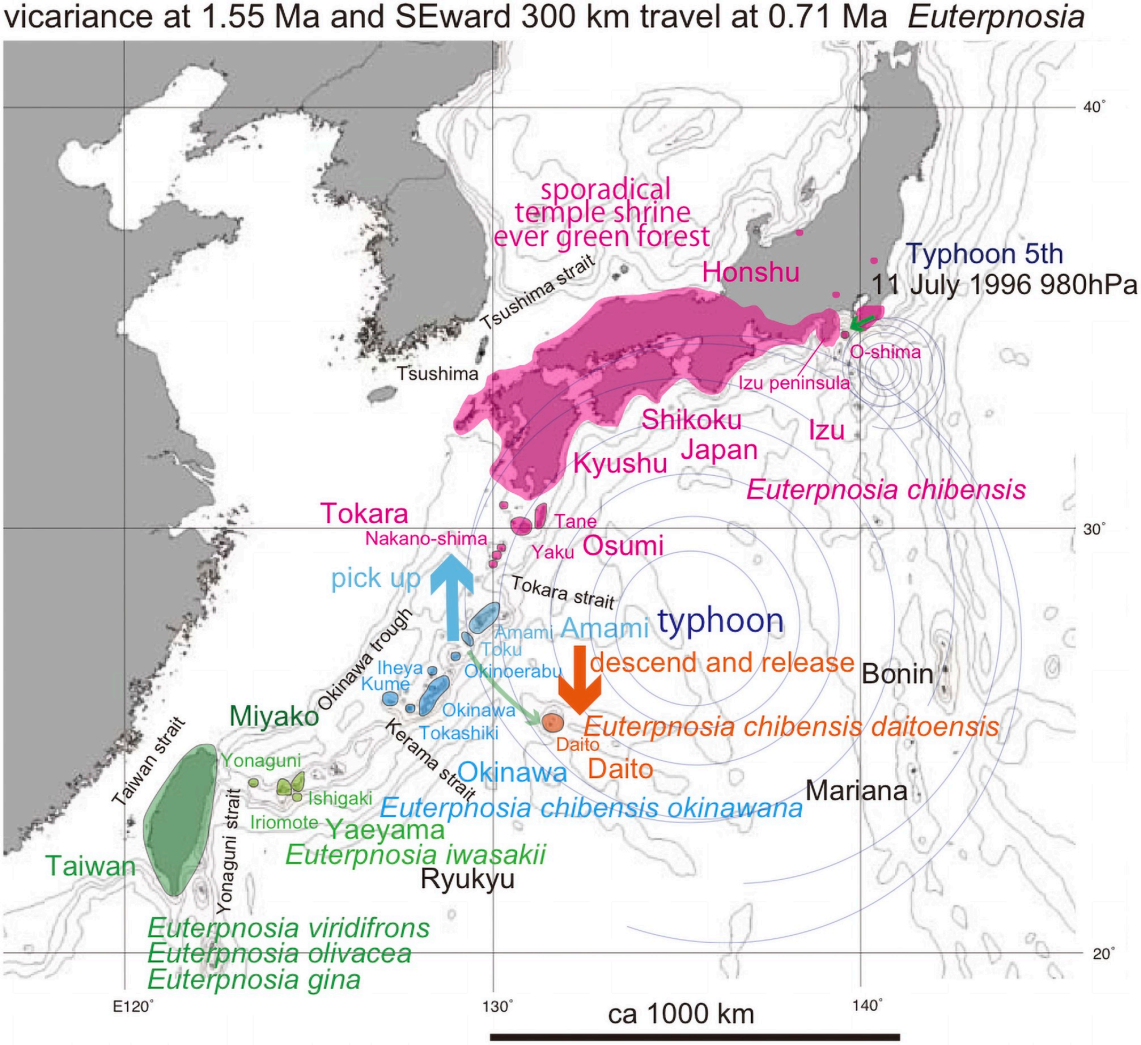
Distribution of *Euterpnosia*. Island populations are depicted by color highlights. See Discussion; Green anticlockwise curved tracks: Typhoon dispersal from Toku to Daito islands (SW part of map), and also from Honshu to O-shima (much shorter path on NE part of map). The bold short arrows schematically (not to scale) show where the typhoon winds picked up cicada (red) and where the same cicada were released (orange). Base map from Vector Map (VMap) Level 0, National Geospatial-Intelligence Agency.

*Euterpnosia* species are distributed from the Himalaya to southern China, Taiwan, Ryukyu islands, and Japan. It diversified into 13 species in Taiwan [7]. In the Ryukyu islands, three endemic species are known from the Amami, Okinawa, and Yaeyama islands, whereas *E*. *chibensis daitoensis* is known from the Daito oceanic islands (Fig 3) off shore (east) of the Ryukyu islands. Under predation pressure by *Hypsipetes amaurotis borodinonis* (brown-eared bulbul) [14], *E*. *c*. *daitoensis* is larking in bush in daytime and not extinct [6]. We will address this apparently anomalous distribution with the analyses in this paper. Note that [14] pointed out that *Pterodroma neglecta* (shearwater) collected on August 3, 1931, on the Minami-Daito island, was dispersed by riding on marginal wind of a typhoon situated to the south at this date, similar to our proposal bellow.

In Japanese main islands, *E*. *chibensis chibensis* is restricted to evergreen forests of southwest Japan, particularly protected forests associated with Japanese shrines. Three isolated habitats constitute the northern limit of this species within Honshu (three small red circles in Fig 3), and the isolated distribution pattern suggests progressive vicariant speciation. In contrast, *E*. *chibensis chibensi*s was also recorded from the Oshima islet, northern end of the Izu oceanic islands, 20 km from the Izu peninsula (Fig 3), since 1998. This may reflect a similar but a smaller-scaled dispersal process comparable to that which may have populated the Daito islands.

### *Meimuna* (Figs 4 and 5)

**Fig 4:**
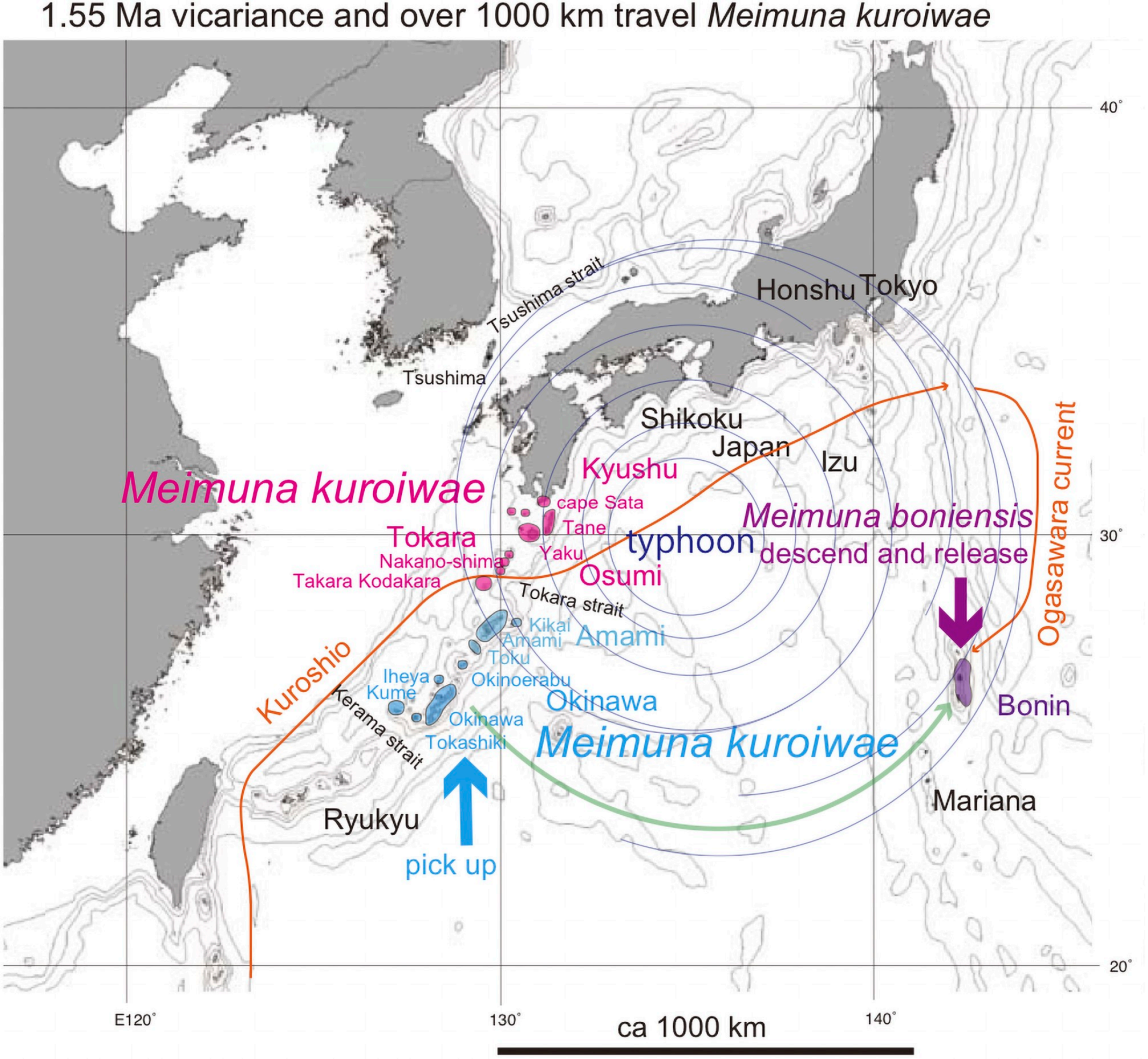
Distribution of *Meimuna kuroiwae*. Island populations are shown by colored highlights. See Discussion; Green anticlockwise curved track: Super typhoon dispersal from Okinawa (blue) to Bonin (purple, *Meimuna boniensis*). The bold short arrows schematically (not to scale) show where the typhoon winds picked up cicada (blue) and where the same cicada were released (purple). The Kuroshio warm current is shown by the thin orange path, and the southward Ogasawara current is also shown. Base map from Vector Map (VMap) Level 0, National Geospatial-Intelligence Agency.

**Fig 5:**
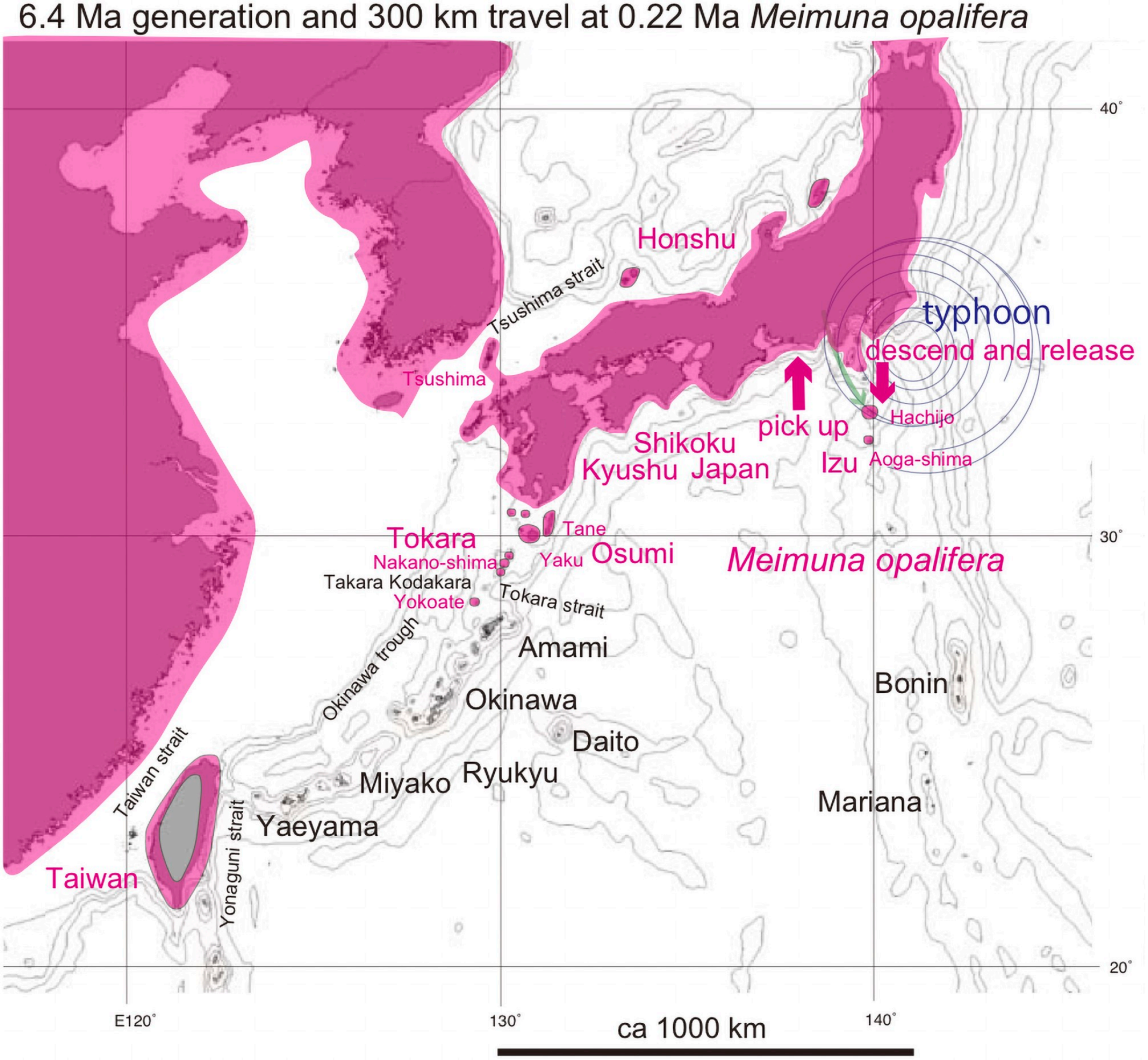
Distribution of *Meimuna opalifera*, that is absent in the Ryukyu islands. See Discussion; Green anticlockwise curved track: Typhoon dispersal from Honshu to the northern Izu islands of Hachijo and Aoga-shima. The bold short arrows schematically (not to scale) show where the typhoon winds picked up cicada (red) and where the same cicada were released (red). Base map from Vector Map (VMap) Level 0.

*Meimuna kuroiwae* has a limited distribution on the Okinawa, Amami, Tokara, and Osumi islands, and at Cape Sata of southernmost Kyushu, and is absent on the Yaeyama islands. Local morphological variation is recognized [6], and vicariant speciation is expected.

*Meimuna boniensis* is known from the Bonin islands, with a distance of 1000 km from the Okinawa islands and also from Tokyo (Fig 4). The morphology and song is similar to *M*. *kuroiwae*, and considered to have recently derived from some of the above localities by transplanting [6]. Because *M*. *boniensis* is rigidly and nationally protected, it is usually impossible to analyze, but the phylogenetic tree was informally shown [15]. Although he did not upload the sequence data into GenBank/DDBJ, *M*. *boniensis* was represented as a sister of *M*. *kuroiwae* in Okinawa, and unrelated to the historic transplanting. Therefore, *M*. *boniensis* may have naturally dispersed from Okinawa to Bonin in pre historic time.

We also analyzed *Meimuna opalifera*, cosmopolitan in Japan, Taiwan, Korea, and China (Fig 5), although this species is lacking and replaced by *M*. *oshimensis* and *M*. *iwasakii*, endemic in the Ryukyu islands (the latter is also known in Taiwan). The cicada’s song on the Osumi islands, Taiwan, and Korea is distinct from that in the Japanese main islands [6], and this may reflect genetic differences so we analyzed this species. This species is also known from the Hachijo-jima and Aoga-shima (southern limit) islands of the Izu oceanic islands, 300 km apart from Tokyo (Fig 5). We noted that the cicada’s song on Hachijo-jima was different from that of the Japanese main islands, so we added a Hachijo specimen to our analyses.

## Analytical methods

### Applied DNA sequence

Mitochondrial COI and nuclear 18S rRNA sequence data from our 92 collected specimens are presented in Table 1. Primers used, amplifications, and sequencing are given in [1]. These sequences were aligned by ClustalW in MEGA X [16]. The COI sequence data comprise 1,534 bp, and the 18S rRNA sequence 874 bp, and the resolution to construct phylogenetic tree and haplotype network was sufficient, as we experienced in the *Platypleura* paper [1].

### Phylogenetic analyses associated with fossil and geological event calibrations by BEAST v1.X

**Fig 6:**
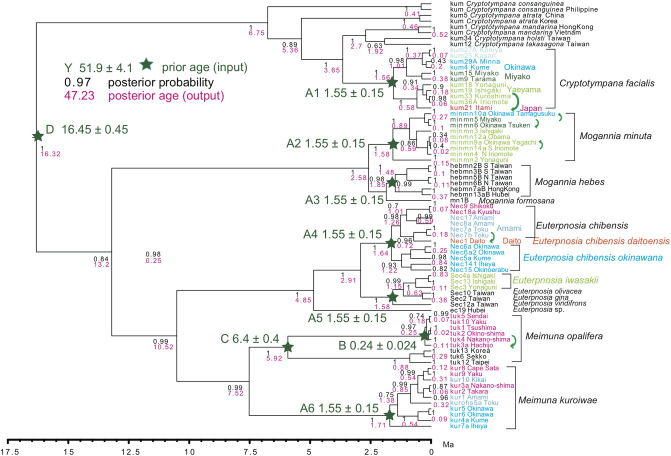
BI tree for *Cryptotympana*, *Euterpnosia*, *Mogannia*, and *Meimuna*. Vicariantly speciated island populations are shown by different colored text labels. Calibration points and dates are shown by green stars and corresponding large font green text labels. Black numbers on each node: posterior probability, red numbers: age (in Ma). The green curved arrows depict dispersal after the vicariance.

A Bayesian inference (BI) tree (Fig 6) was constructed using the software BEAST v.1X [17], running BEAUti, BEAST, TreeAnnotator, and FigTree, in ascending order. Before operating the BEAST software, the BEAGLE Library must be downloaded.

See “BEAST v1.X tutorial, in a case of four cicada genera" at: dx.doi.org/10.17504/protocols.io.bq6mmzc6.

Calibrations points are shown on Fig 6, and these dates were input in “Priors” in BEAUti; they are summarized below. Corresponding ingroup species were included in ingroup taxa by “Taxon Set” on the “Taxa” screen in BEAUti.

Calibration point A (A1 to A6) is after our geological event calibration that adopts a 1.55 Ma date based on multiple biostratigraphic and supporting radio-isotopic dates connected to various geologic relationships in the Ryukyu islands region [18]. This geologic event calibration was also used in a previous study of *Platypleura* cicadas by our group [1].

Calibration point B: Hachijo oceanic island is a part of the Izu volcanic arc, and we recently estimated the emergent time of Hachijo as an island at 0.24 Ma [19]. This date is applicable for *Meimuna opalifera* on the Hachijo-jima and the Japan continental islands.

Calibration point C: Crown *Meimuna opalifera*: Fossil *M*. *protopalifera* was found from the Itamuro Formation, Tochigi, Japan [20, 21]), and the fission track age of the correlative terrestrial strata of the Nashino Formation, Sendai, is 6.4 ± 0.4 Ma [22].

Calibration point D: Crown *Cryptotympana*: Fossil *C*. *incasa* and *C*. *miocenica* were found from Shanwang, Shandong, China [23], and these strata are considered to be time correlative to the European MN5 mammalian stage (16.45 ± 0.45 Ma) [24].

### Haplotype network analyses by Network

In DnaSP 6.12.13 [25], sequence files such as fasta files were converted into rdf files.

In the platform software of Network 10, inputting the above rdf file, calculation and graphic outputting was performed.

For graphic explanation of the operation of these softwares, see the “Network tutorial” at:

http://kawaosombgi.livedoor.blog/archives/21400217.html

## Results

### BI tree (Fig 6)

The BI tree consists of the *Cryptotympana*, *Mogannia*, *Euterpnosia*, and *Meimuna* clades. The *Cryptotympana* clade consists of *C*. *facialis* and the remaining five species, and *C*. *facialis* is not a sister of *C*. *takasagona*. The *Meimuna* clade consists of *M*. *opalifera* and *M*. *kuroiwae* clades. *Cryptotympana facialis*, *Mogannia minuta*, *Mogannia hebes*, *Euterpnosia chibensis*, *Euterpnosia iwasakii* + Taiwan *Euterpnosia*, *Meimuna kuroiwae* were simultaneously differentiated at 1.55 Ma as calibrated by this date.

For *Cryptotympana facialis*, note that the sequence data of kum25 Kasari (Amami) = kum26 Naze (Amami) = kum10 Toku Amagi = kum28A Toku Amagi = kum28B Toku Amagi = kum2 Yoron = kum30 Okinawa Kunigami = kum31 Okinawa Nago = kum20 Okinawa Yaese (Table 1), and the sequence data are common on the Okinawa main island and the Amami islands, except for kum27AB Koniya. The Okinawa (+ Amami) population is a sister of the Miyako population, but the northernmost Japan (+ Osumi and Tokara) population is a sister of the southernmost Yaeyama population (Fig 1).

For *C*. *facialis*, note that kum21 Itami = kum14 Kagoshima = kum23A Tane = kum22A Yaku = kum8 Nakano-shima = kum13 Takara = kum24A Kodakara (Table 1), and the sequence data are regionally the same in the Japanese main islands and the Osumi and Tokara islands. The COI-5P sequence data found in GenBank/DDBJ are the same as our data for a total of 106 specimens collected from Honshu and Kyushu, although four specimens differ one base pair relative to these 106 specimens.

For *Mogannia minuta*, sequence data of minmn9a Okinawa Yagachi (Fig 2 inset) is identical to minmn14a S Iriomote (Yaeyama). The sequence data of minmn6 Okinawa Tsuken (new habitat of Okinawa-jima; Fig 2 inset) is similar to minmn5 Miyako, but more differentiated for minmn10a Okinawa Tamagusuku (specimen collected at the original habitat in southern Okinawa-jima; Fig 2 inset).

For *Mogannia hebes*, the northern Taiwan population is a sister of the Chinese mainland population west of the Taiwan strait, and the southern Taiwan population is a sister of northern Taiwan + China population.

For *Euterpnosia chibensis*, N-ec9 Shikoku differs by only one base pair from N-ec18a Kyushu, and this Japan population is a sister of the Amami population. Nec1 Daito (*E*. *chibensis daitoensis*) is a sister of Nec7ab Toku, and these have a sister relationship to the Japan + Amami population. *E*. *chibensis* in these areas is a sister of *E*. *chibensis okinawana* on Okinawa. *Euterpnosia iwasakii* (Yaeyama) is morphologically similar to *E*. *viridifrons* (Taiwan) [6], but a sister of *E*. *olivacea* + *E*. *gina*.

For *Meimuna kuroiwae*, the northern population including Cape Sata, Osumi, Tokara, and Amami islands, is a sister of the southern population of Okinawa, but kur7a Iheya is strongly differentiated.

*Meimuna opalifera* on the Japan islands and northern Tokara islands was mildly or not differentiated within these areas, but moderately differentiated on the Hachijo island relative to the others (= sister of the Japan population). The Japan population including the Hachijo island is a sister of the Korea + China + Taiwan populations, and tuk12 Taipei is strongly differentiated relative to the others. *M*. *opalifera* is absent from the main part of the Ryukyu islands.

### Haplotype network

#### *Cryptotympana* (Fig 7)

**Fig 7:**
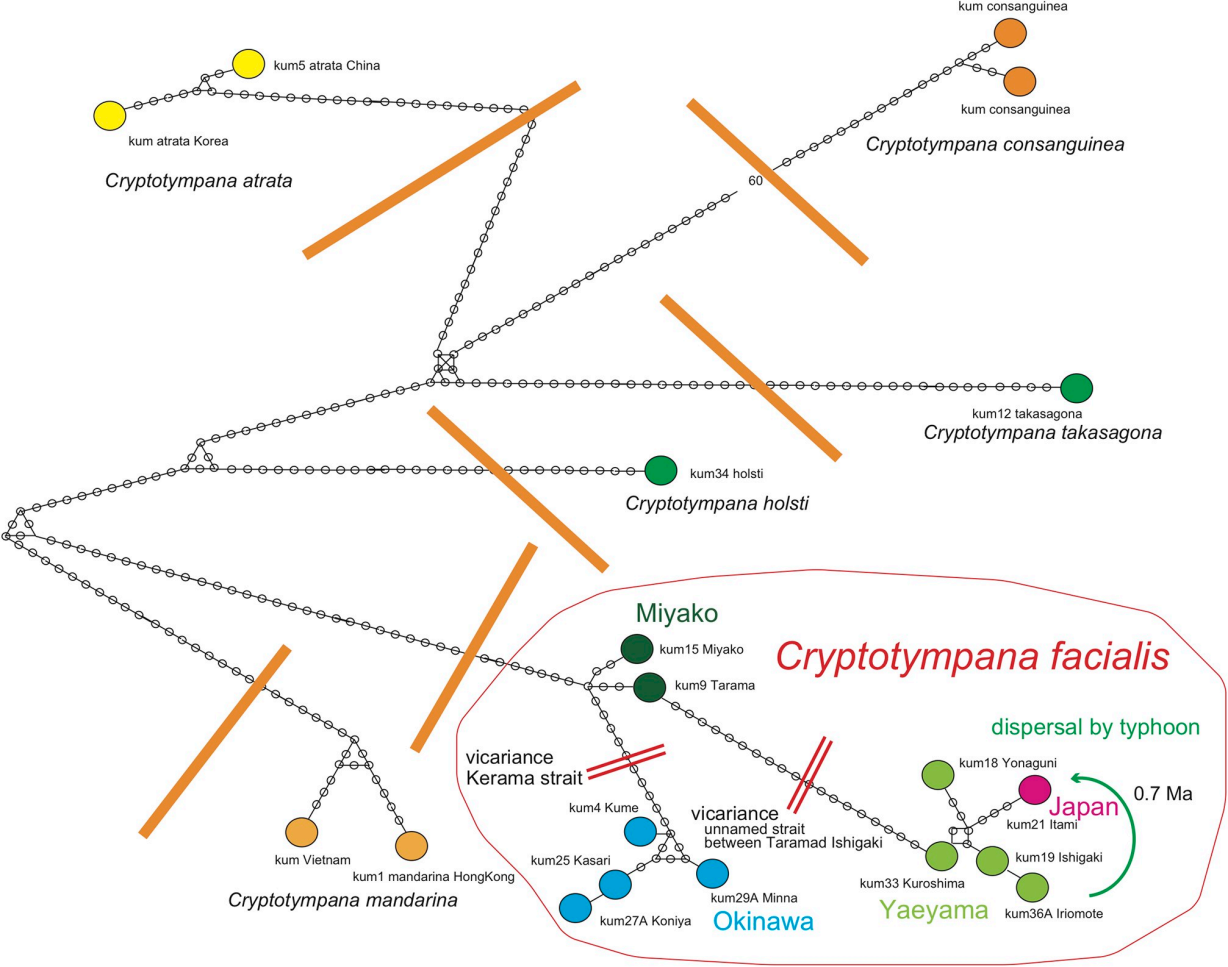
Haplotype network of *Cryptotympana*. *C*. *facialis* was vicariantly speciated due to isolation of island groups of Okinawa, Miyako, and Yaeyama, separated by major straits (red double line) formed since 1.55 Ma, and three island group haplotypes or populations were formed. However, the Japan haplotype was included in the Yaeyama haplotype network, suggesting a long-distance dispersal (green curved arrow) from Yaeyama (southern end of Ryukyu) to Japan at 0.58 Ma (Fig 6), crossing Miyako and Okinawa. Orange heavy line: *C*. *facialis* is separated by the other *Cryptotympana* species including *C*. *takasagona*.

The six analyzed species contain many genetically distinct interspecies haplotypes, and *Cryptotympana facialis* is genetically distinct from *C*. *takasagona*. Species level differentiation is recognized in *C*. *atrata* in Korea and China, *C*. *mandarina* in Hong Kong and Vietnam, and *C*. *consanguinea* on Mindanao of the Philippines.

Three areal haplotypes of the Okinawa, Miyako, and Yaeyama islands, from north to south, respectively, are recognized for *Cryptotympana facialis*, and these haplotypes, separated by major straits such as the Kerama strait (Fig 1). The northernmost Japanese haplotype, however, is included in the southernmost Yaeyama haplotype, as is not an independent haplotype. In addition, the Japanese haplotype kum21 Itami contains the same base pair haplotype as the Osumi and Tokara haplotypes and kum22A Yaku and kum24A Kodakara + 102 haplotypes of Honshu and Kyushu, and the 4 haolotypes that differ by one base pair (red colored region in Fig 1).

#### *Mogannia* (Fig 8)

**Fig 8:**
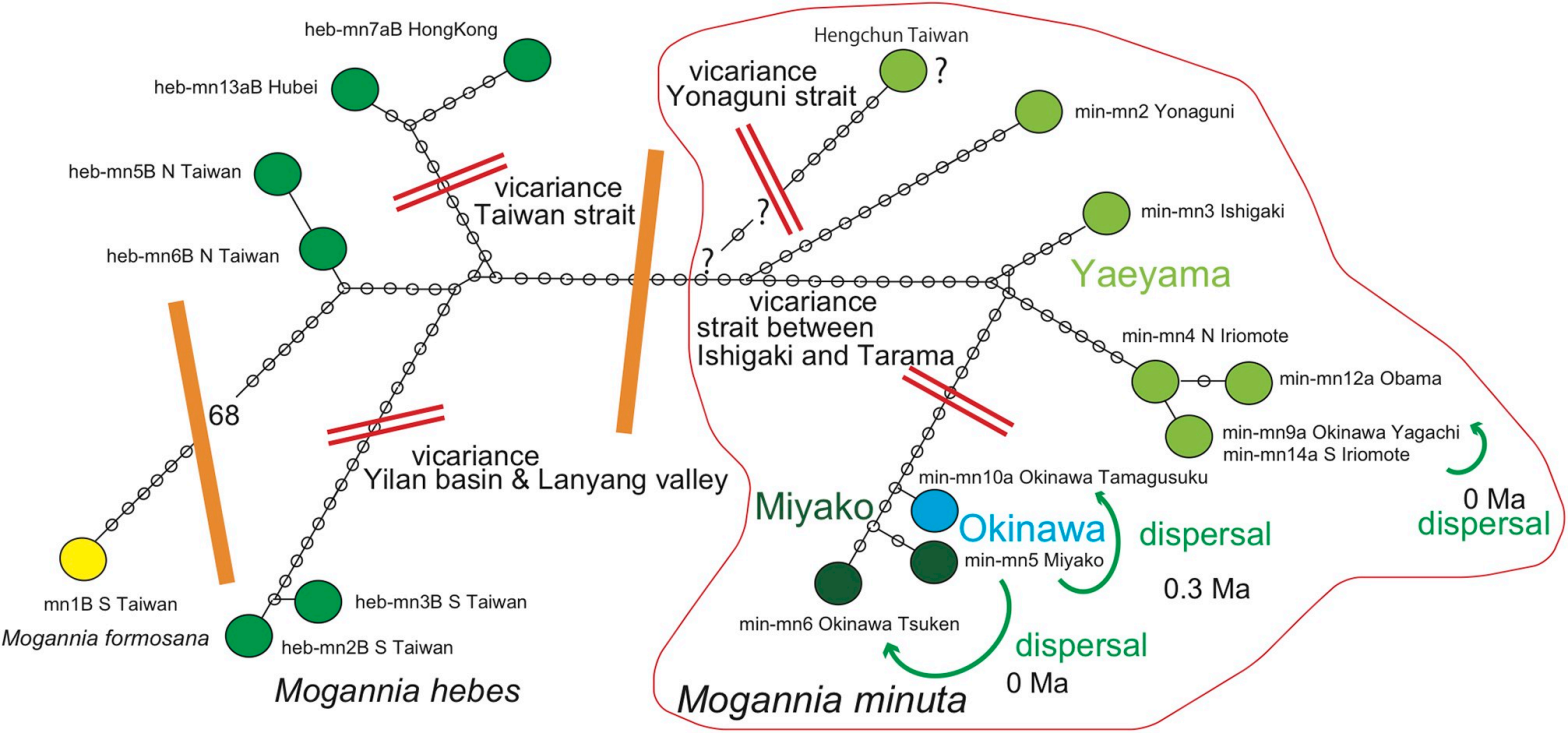
Haplotype network of *Mogannia*. On each of Yaeyama islands, *Mogannia minuta* constitutes the distinct haplotype, reflecting vicariant speciation since 1.55 Ma. However, this species was recently recorded from Yagachi-jima (Fig 2 inset), northern Okinawa, and the haplotype is identical to the haplotype of S (southern) Iriomote-jima, suggesting recent long-distance dispersal. The Miyako and the original southern Okinawa populations (Fig 2 inset) were included in the same Miyako haplotype network, and the southern Okinawa population originated from the Miyako population, dispersed at 0.27 Ma (Fig 6). The network of the recently recorded specimens in southern and central Okinawa such as Tsuken (Fig 2 inset) suggests recent dispersal also from the Miyako islands. Red double line: barrier for vicariance. Orange heavy line: *M*. *hebes* is separated by *M*. *minuta*, but genetically closely related.

A haplotype network comprises *Mogannia minuta* and *Mogannia hebes*. *Mogannia formosana* is a distinct haplotype.

The *Mogannia minuta* network consists of Yaeyama and Miyako-Okinawa haplotypes. An expected Taiwan haplotype is shown on this Fig 8. A haplotype consists of the same sequence as min-mn14a S Iriomote and min-mn9a Okinawa Yagachi (Fig 2 inset). In the Miyako-Okinawa network, min-mn5 Miyako is similar to min-mn6 Okinawa Tsuken, and min-mn10a Okinawa Tamagusuku (original habitat).

The *Mogannia hebes* network consists of Chinese, northern Taiwan, and southern Taiwan haplotypes, differentiated from one another.

#### *Euterpnosia* (Fig 9)

**Fig 9:**
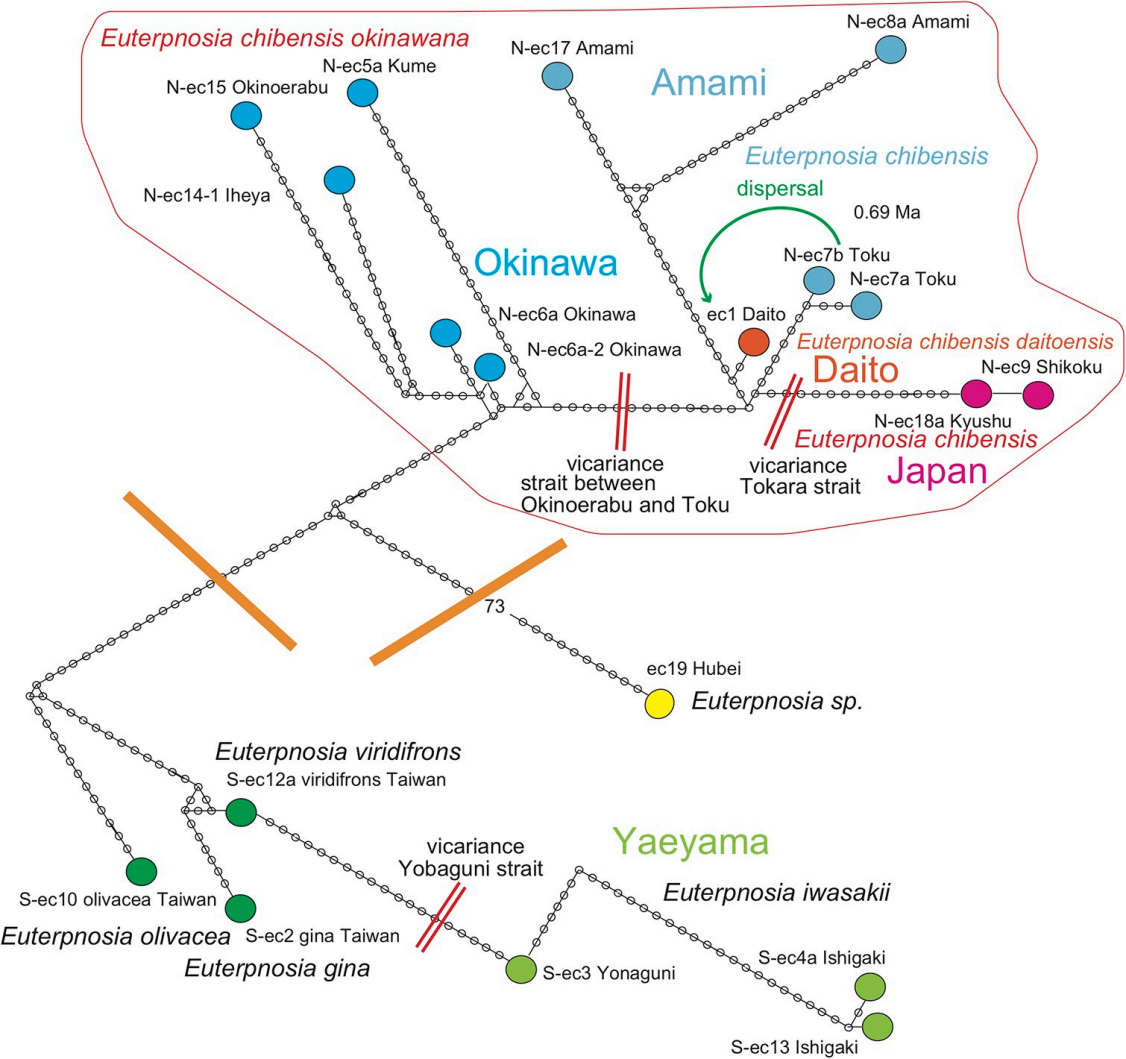
Haplotype network of *Euterpnosia*. The haplotypes were divided into *E*. *chibensis okinawana* (Okinawa) and *E*. *chibensis* networks, and the latter divided into the Amami and Japan networks, reflecting the 1.55 Ma event of vicariant speciation acted on the Ryukyu islands. However, *E*. *chibensis daitoensis* from the island of Daito was included in the Amami network, showing particularly close resemblance to the Toku network, suggesting that the Daito population was dispersed from the Tokuno-shima island at 0.72 Ma (Fig 6).

Haplotype network consists of northern Japan-Amami-Okinawa and southern Yaeyama-Taiwan haplotypes. The Chinese haplotype is distinct from the remaining haplotypes.

*Euterpnosia chibensis daitoensis* of ec1 Daito is close to *E*. *chibensis* of N-ec7ab Toku. *E*. *chibensis* haplotypes as a whole comprise a network, whereas the Japanese haplotypes of N-ec9 Shikoku and N-ec18a Kyushu constitutes a distinct network from the Amami network. E. *chibensis okinawana* also constitutes a distinct network.

S-ec3 Yonaguni of *E*. *iwasakii* is close to S-ec12a of *E*. *viridifrons*, but S-ec2 of *E*. *gina* and S-ec10 of *E*. *olivacea* constitute a single Taiwan network with S-ec12a.

#### *Meimuna* (Fig 10)

**Fig 10:**
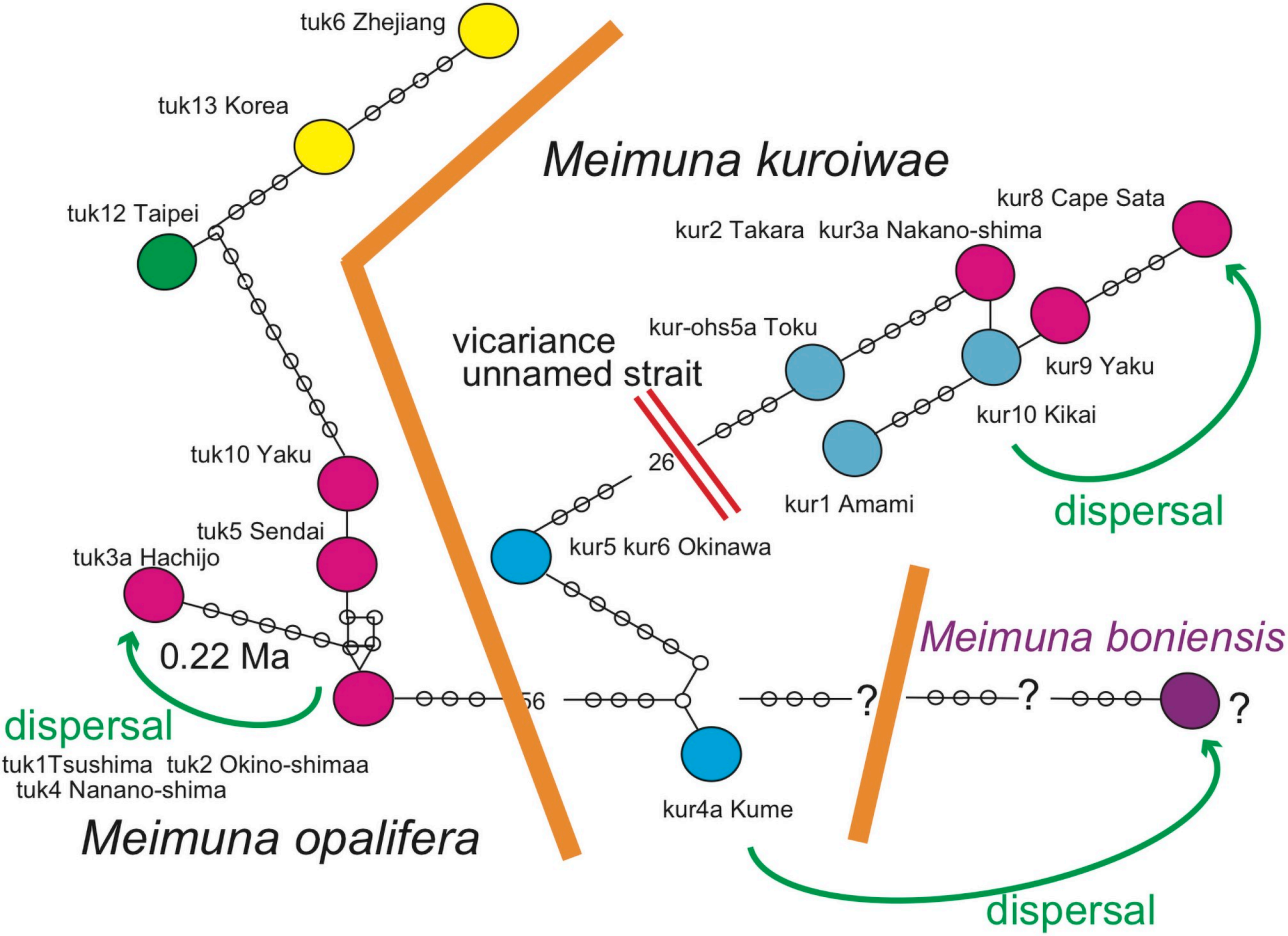
The haplotype network of *Meimuna*. *Meimuna kuroiwae* and *M*. *opalifera* constitute different networks. The *M*. *kuroiwae* network divided into the Okinawa and Amami-Tokara-Osumi-Kyushu networks, and the population of the southern end Kyushu may have been derived by dispersal from the southern islands. *Meimuna boniensis* may have been dispersed from Okinawa in ancient times (Nagata, 2019).

*Meimuna kuroiwae* network consists of the Cape Sata-Osumi-Tokara-Amami and Okinawa haplotypes. Haplotype of southern kur10 Kikai is connected with kur9 Yaku with one base substitution, and then northernmost kur8 Cape Sata with four base substitutions. The expected *Meimuna boniensis* haplotype is connected with the Okinawa haplotype [15].

*Meimuna opalifera* network consists of the Japan and Korea-China-Taiwan haplotypes. The Japanese haplotypes are not differentiated, and a haplotype consists of the same sequence of tuk1 Tsushima, tuk2 Okino-shimaa, and tuk4 Nanano-shima. However, tuk3a Hachijo is differentiated relative to the remaining Japan haplotypes.

## Discussion

### Vicariance due to separation by seaways

In Fig 6, *Cryptotympana facialis*, *Mogannia minuta*, *Mogannia hebes*, *Euterpnosia chibensis*, *Euterpnosia iwasakii* + Taiwan *Euterpnosia*, *Meimuna kuroiwae* were simultaneously differentiated at 1.55 Ma, which suggests vicariant speciation acted on the most recent common ancestors of these ingroup species. As we showed for *Platypleura* cicada [1], the differentiation trigger was the physical separation of islands from the Chinese mainland as a result of with rifting (by sea-floor spreading) of the Okinawa trough that started at 1.55 Ma [18].

In the haplotype network of *Cryptotympana facialis* (Fig 7), *Mogannia minuta* and *Mogannia hebes* (Fig 8), *Euterpnosia chibensis* and *Euterpnosia iwasakii* + Taiwan *Euterpnosia* (Fig 9), and *Meimuna kuroiwae* (Fig 10), red double line indicates vicariance between island groups within a single species (whereas *E*. *iwasakii* + Taiwan *Euterpnosia* is an extra species level), although orange line indicates species level classification with the above exception. Such networks separated by red double lines in these figures are geographically separated by major seaways also shown in Figs 1–4. Classification into each haplotype in these haplotype networks (Figs 7–10) reflects milder vicariance separated by minor seaways, although sporadically connected with each other during periods of lower sea level during glacial episodes [1].

Because cicada nymphs live underground for two to five years (*Meimuna opalifera*: exceptionally one to two years [6]; c.f., *Magicicada*: 13-year and 17-year in eastern North America [26]), nymphs are unable to cross the seaways, so cicadas tend to be affected by vicariant speciation, as reflected in the vicariance of analyzed cicada species.

### Natural and artificial dispersals

#### Reason for dispersal

Some network patterns are explained by occasional dispersal, even where severe vicariance acted on these cicadas. Dispersal may be natural or artificial (anthropogenic), and modern or ancient for natural dispersal.

In the *Cryptotympana facialis* network (Fig 7), the Japanese haplotype constitutes a part of the Yaeyama haplotype, and the mild differentiation between them is hard to explain by simple vicariance, in contrast to the case of the Yaeyama vs Miyako haplotypes. We suggest dispersal of the Japanese population from the Yaeyama population (green arrow in Fig 7), and the dispersal date has an estimated node age of 0.58 Ma between the Japan and Yaeyama clades in the dated BI tree (Fig 6). Note that the haplotype shows a simultaneous diffusional pattern (Fig 7).

The original habitat of *Mogannia minuta* was on the Chinen peninsula of southern Okinawa (Fig 2 inset), but it spread northward since 2004 (Sasaki, 2011; record in websites since 2012). The haplotype network consists of Yaeyama and Miyaki-Okinawa (Fig 8), but sequence data or haplotype of min-mn9a Okinawa Yagachi (Fig 2 inset; Note that the new habitat is restricted in this small island) is the same as min-mn14a S Iriomote, and the Yagachi population might have dispersed from the southern Iriomote-jima before 2012 (Yagachi record is since 2012). Note that northern Irionote haplotype of min-mn4 N Iriomote differs by one base pair from S Iriomote (Fig 8). The new habitat haplotype of min-mn6 Okinawa Tsuken (Fig 2 inset) is similar to min-mn5 Miyako, and also dispersed from the Miyako islands before 2012 (Table 1), rather than from the original habitat on the Chinen peninsula. The Miyako islands consist of the Miyako-main, Shimoji, and Tarama islands. Whereas we did not analyze Shimoji and Tarama island specimens, we expect that they would be the same as min-mn6 Okinawa Tsuken. The haplotype of min-mn10a Okinawa Tamagusuku on the Chinen peninsula (Fig 2 inset) is also similar to min-mn5 Miyako, although more differentiated than min-mn6 Okinawa Tsuken. *Mogannia minuta* originally of southern Okinawa was dispersed at about 0.27 Ma, estimated by the node age between the Miyako and the original Okinawa clades in the dated BI tree (Fig 6).

The Daito oceanic islands were originally a remnant volcanic arc separated from the Izu volcanic arc by a series of back arc spreading events, including the formation Shikoku basin, concluding at 15 Ma. Thereafter the islands such as the Daito islands on the Philippine Sea plate have approached the Ryukyu continental arc, and have begun to collide with the trench/subduction zone or will collide in the future. On the Daito islands, far from the continental landmass, cicada may have originally been absent. Haplotype of ec1 Daito is close to N-ec7ab Toku (Fig 9), and the Daito *Euterpnosia* population may have dispersed from the Tokuno-shima island. The dispersal date is an estimated node age of 0.72 Ma between the Daito and Toku clades in the dated BI tree (Fig 6).

Hachijo oceanic island is a part of the Izu volcanic arc, its *Meimuna opalifera* haplotype is included in the Japanese network (Fig 10), and should have dispersed from the Japan main islands. The dispersal date is an estimated node age of 0.25 Ma between the Hachijo and the resting Japan clades in the dated BI tree (Fig 6). As noted above, we recently estimated the emergent time of Hachijo as an island at 0.24 Ma [19]. If the *Meimuna boniensis* haplotype is connected to the Okinawa haplotype, as [15] inferred, the Bonin population may have dispersed from Okinawa, and the date was estimated at 1.4 Ma [15]. The emergent time of Bonin as an island is not geologically estimated.

#### Dispersal by typhoon as a viable mechanism

*Recent records and observations*. Typhoon marginal winds rotate counter-clockwise at lower altitudes in the northern hemisphere, including Ryukyu islands, affected by Coriolis force. The marginal wind is strongest east of the eye, reinforced by the westerlies. In the eye, the warm rising air also rotates counter-clockwise, but radial flow from the eye at more than 10,000 m altitude is clockwise also affected by Coriolis force. A super typhoon is defined by an area of more than 800 km in radius affected by wind velocity of >15 m/s.

Flying insects can be carried by typhoon marginal winds. If the velocity is 15 m/s and distance between pick up and release points is 1000 km, the transport time is only 18.5 hours, and the insects may be able to survive during the travel.

**Fig 11:**
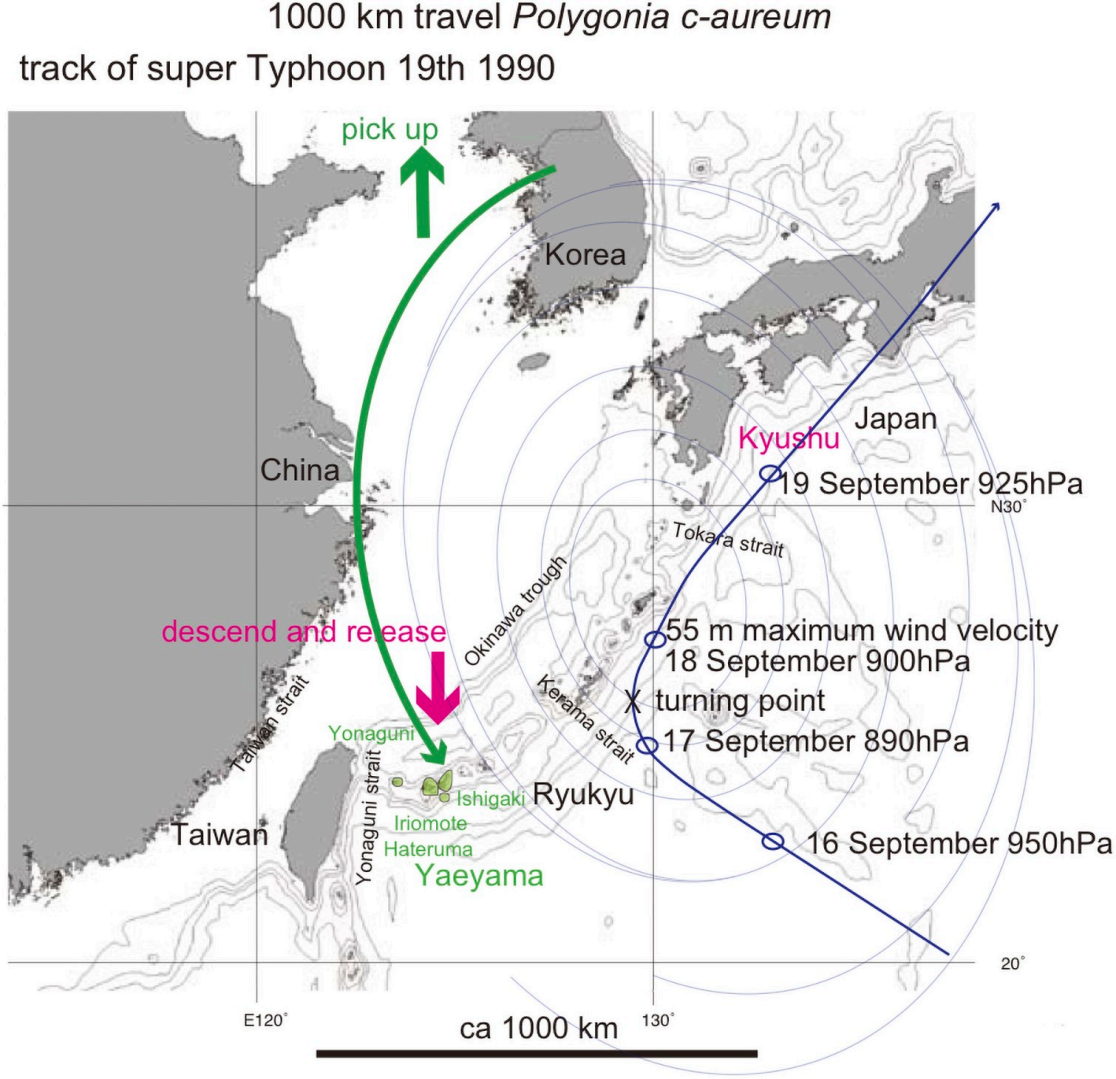
Track of super Typhoon 19, 1990 (blue fine curved path), and 1000 km inferred transport of *Polygonia c-aureum* by typhoon winds (green curved arrow). The bold short arrows schematically (not to scale) show where the typhoon winds picked up cicada (green) and where the same cicada were released (red). Base map from Vector Map (VMap) Level 0, National Geospatial-Intelligence Agency.

A modern example of live insects transported by typhoon marginal wind was shown by [27]. *Polygonia c-aureum* (a nymphalid butterfly) is absent from the Ryukyu islands. However many specimens of its autumn form were collected as stray butterflies on Ishigaki (2 exs), Iriomote (28 exs), Hateruma (1 female), Yonaguni (1 ex), and Okinawa (1 male) islands in September 19 ~ 23, 1990. The butterflies (not exclusively a single gravid female but multiple) were probably transported by southward marginal wind of the super typhoon 19, 1990, from temperate Korea or northern China to these subtropical islands (Fig 11). Typhoon generally tracks around the western margin of the Pacific High, northwestward in lower latitude affected by the trade wind, and then northeastward affected by the westerlies. The typhoon tends to be stationary at the turning point shown in Fig 11.

It was also pointed out that the northeastward wind around the Pacific High might have carried stray butterflies from southern islands such as Taiwan and Philippine [27]. The records of a stray butterfly or butterflies were mostly after typhoon attack, and newly generated butterflies derived from gravid female(s) were reported [28]. Subtropical butterflies were reported to have extended habitats northeastward in the Ryukyu and Japan islands since 1950s, mostly by the wind dispersal including typhoon [29]. However, some southern butterflies have been expanding their geographic ranges northward as a result of global warming in recent decades [30].

Flying insects can be carried in the raising air at the typhoon eye after being transported by the marginal wind, and as the typhoon abates, the insects descend to the ground at this release point, but these may be somewhat exceptional cases.

**Fig 12:**
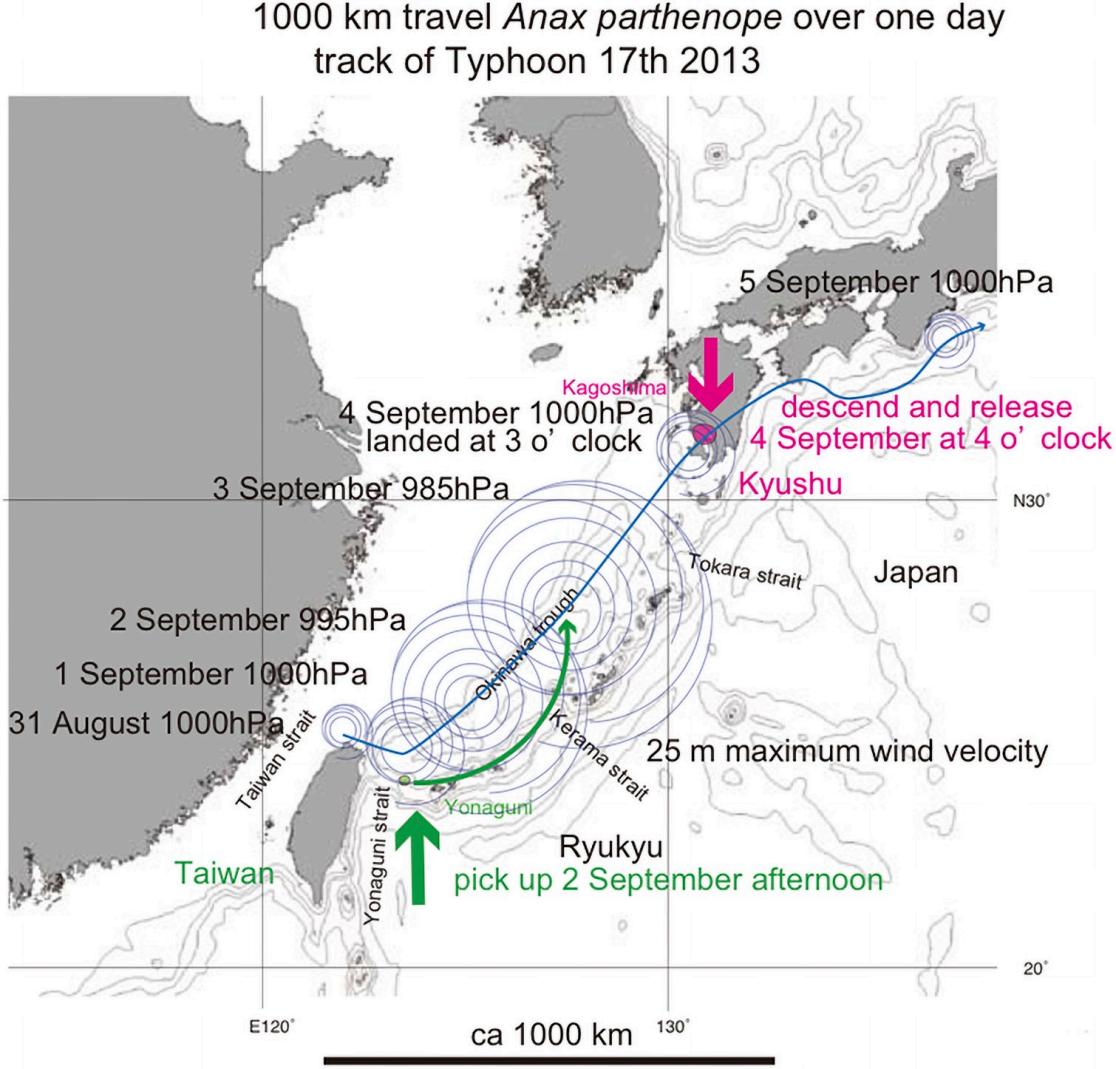
Track of Typhoon 17, 2013 (fine blue sinuous path), and hypothetical 1000 km transport of *Anax parthenope* over one day by typhoon winds (green curved anticlockwise path). The bold short arrows schematically (not to scale) show where the typhoon winds picked up cicada (green) and where the same cicada were released (red). Base map from Vector Map (VMap) Level 0, National Geospatial-Intelligence Agency.

One such exception was reported [31]. Typhoon 17, 2013, was generated offshore of northern Taiwan as a tropical cyclone at 2100 h on 31 August, migrated to the west of Yonaguni-jima, path along the west shore of the Ryukyu islands, landed on the Kagoshima (southernmost Kyushu) coast at 0300h on 4 September, diminished in strength and lost its eye over the Kagoshima city area, and declined to a temperate cyclone (Fig 12). Many *Anax parthenope* were observed on Yonaguni-jima island on 2 September but vanished on 3 September, probably carried away by the marginal winds of typhoon 17, whose track was west of the Okinawa and then the Amami islands at these dates. Many active *A*. *parthenope* were later observed in the Kagoshima city area. More than 90 reports of this dragonfly mating and egg-laying were made by Kagoshima citizens to the Kagoshima Insect Society from the early morning of 4 September. These observation suggest that multiple *A*. *parthenope* were transported by the typhoon 17, with a distance over 1000 km from the Yonaguni-jima island to the Kagoshima city in 24 hours.

Our phylogenetic study of another dragonfly of *Chlorogomphus* indicated recent migration from the Amami islands to Shikoku, Japan, as a result of transport by typhoon winds [5]. *Chlorogomphus* has a habit crowing in the sky using updraft, and may have own ability of gliding long distance [29]. Note that super typhoon are expected to have been generated in the East China Sea after 1.55 Ma, but not before that. This is because the sea floor spreading of that formed the Okinawa trough thereafter, typhoons tracks in this region would have made land fall and lost energy, rather than continuing northward to Japan [5].

*Cryptotympana facialis*. Counterclockwise marginal wind of super typhoon with a turning point in the mid East China Sea would have transported *Cryptotympana facialis* from Yaeyama to elsewhere Japan-Osumi-Tokara, passing through the Okinawa and Amami islands (Fig 1; 0.58 Ma: Fig 6).

Owing to the large size of *C*. *facialis* compared to the minimal size of *Mogannia minuta*, one may consider whether this cicada can be safely transported for long distance before the release. The terminal settling velocity of tephra (volcanic ash) particles were estimated by considering Stokes’s law and Suzuki’s low with Cunningham correction [32]. For a case of density = 1,000 kg/m^3^ (= water; cicada, ca. 700 kg/ m^3^), and radius of 10 mm (= *Cryptotympana facialis*; 1/3 for *Mogannia minuta*), the settling velocity is less than 10 m/s (7 m/s for the former; 3.3 x 0.7 = 2.3 m/s for the latter), but may be much slower considering the irregular shape and large wings of cicadas compared to idealized spherical or cubic shapes. Compared to the lateral air transport for super typhoon more than 15 m/s, cicadas, like violcanic ashes, may be able to drift in air and fly long distance before landing.

*Mogannia minuta*. Considering the sighting of this species at its dispersed location in 2004 [4] and the 2-year nymph period, the typhoon that transported mated female(s) was probably pre-2002, and considering the emergent season, the track time should have been May to July. Annual typhoon tracks are recorded on the Japan Meteorological Agency (https://www.jma.go.jp/jma/indexe.html) and related websites. Using these records we may evaluate the possible typhoons that transported the cicada(s).

**Fig 13:**
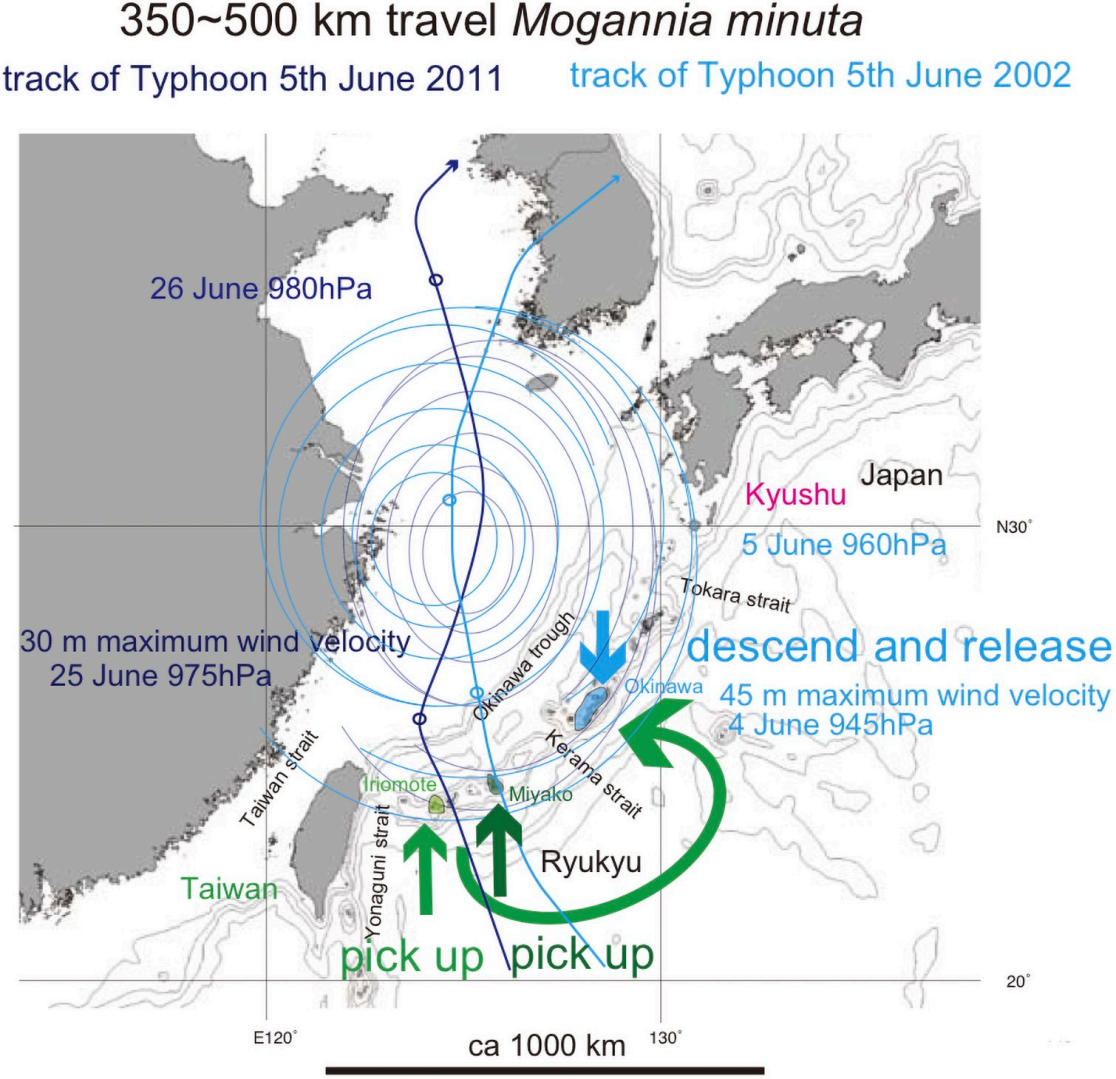
Track of track of Typhoon 5, June 2002 (medium blue curved path) and track of Typhoon 5, June 2011 (very dark blue sinuous path), and hypothetical 350~500 km transport *Mogannia minuta* by the marginal typhoon winds (green curved path). The bold short arrows schematically (not to scale) show where the typhoon winds picked up cicada (green and dark green) and where the same cicada were released (blue). Base map from Vector Map (VMap) Level 0.

Only Typhoon 5, of early June 2002 (Fig 13) appears viable to have transported the cicada by marginal wind. For comparison, of potential post-2004 tracks, only Typhoon 5, late June 2011 (Fig 13) had a track route with the potential for a similar marginal wind dispersal for *M*. *minuta*. That specific typhoon had the potential to disperse from Iriomote-jima as the Yagachi population found after 2012, but not the other Okinawa population dispersed from the Miyako islands (Fig 2 inset), because late June is still the adult season on Iriomote-jima but not on the Miyako islands.

**Fig 14:**
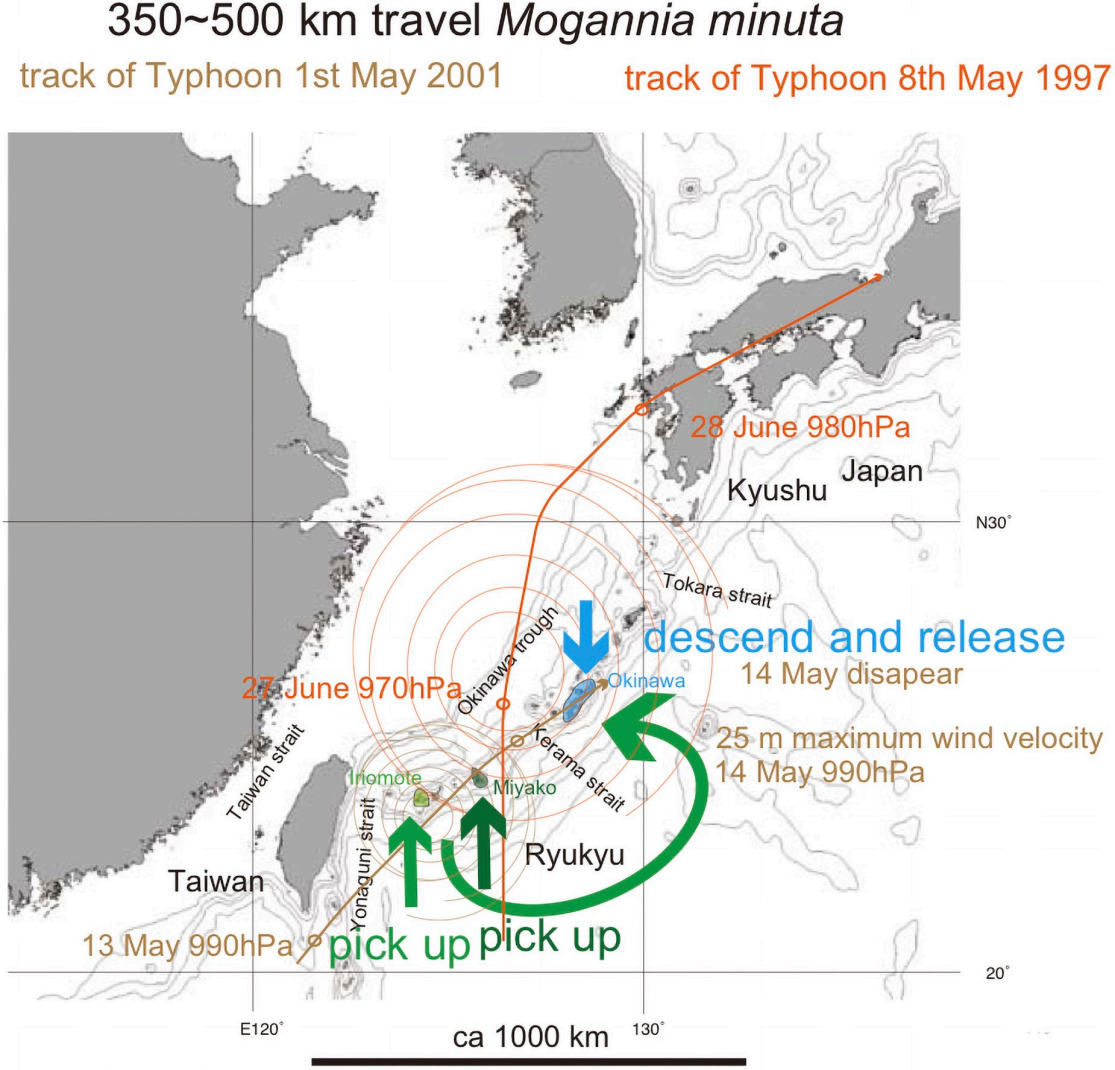
Track of Typhoon 1, May 2001 (brown curved path), that may have transported and then released *Mogannia minuta* on 14 May when it was lost energy west of Okinawa. The track of Typhoon 8, May 1997 (orange curved path), is also shown, because this typhoon also followed a path that may have transported *M*. *minuta* from the southern Ryukyu islands to Okinawa. The green path depicts this hypothetical transport. The bold short arrows schematically (not to scale) show where the typhoon winds picked up cicada (green and dark green) and where the same cicada were released (blue). Base map from Vector Map (VMap) Level 0, National Geospatial-Intelligence Agency.

Typhoon 1, May 2001 (Fig 14), that passed along the southern Ryukyu islands, over the Yaeyama and then Miyako islands on 13 May, lost energy east of Okinawa island on 14 May, and may have released the cicada. In Fig 14, we also show the track of Typhoon 8 May 1997, another potential typhoon that may have transported *M*. *minuta* in its marginal winds.

*Mogannia minuta* in the original habitat of southern Okinawa (Fig 2 inset) may have been dispersed 350 km from Miyako at 0.27 Ma (Fig 6) by marginal wind of typhoon with a turning point in the southern East China Sea (Fig 2), or by a track similar to that of Typhoon 1, May 2001 (Fig 14).

*Euterpnosia chibensis daitoensis*. This endemic species on the Daito islands is a sister of *Euterpnosia chibensis* on Tokuno-shima island, and may have dispersed from Tokuno-shima at 0.72 Ma (Fig 6). If a turning point of super typhoon was in the central Philippine Sea, the southeastward marginal wind west of the center could transport *Euterpnosia* of Toku toward Daito (Fig 3).

*Euterpnosia chibensis* on the O-shima island of the northernmost Izu islands might have recently (pre 1998) dispersed from Honshu. Typhoon 5, July 1996 may have transported *E*. *chibensis* on the Boso peninsula (Honshu) toward O-shima (Fig 3).

*Meimuna*. *Meimuna boniensis* endemic on the Bonin islands is a sister of *M*. *kuroiwae* on the continental Okinawa islands [15], and should have dispersed from Okinawa in pre-historic time, crossing the Pacific ocean. If a turning point of super typhoon was offshore southwest Japan, the eastward marginal wind south of the center may have transported *Meimuna* of Okinawa toward the Bonin islands (Fig 4).

*Meimuna opalifera* habitat extends southward to the Hachijo and Aoga-shima islands, of the central Izu islands (Fig 5). Note that habitat of *Cryptotympana facialis* is limited to Miyake island (Fig 1), suggesting less flying ability for *C*. *facialis*. The southward dispersal at 0.25 Ma (Fig 6) could be by southward marginal wind of typhoon offshore the Boso peninsula (Fig 5).

*Meimuna opalifera* is also known from the northern Tokara islands, but also on the uninhabited Yokoate island, south of the Tokara strait (Fig 5). It is absent on the southern Tokara islands of Takara and Kodakara, so the Yokoate population may have been dispersed from the northern Tokara islands by transport by typhoon marginal winds.

#### Rafting of egg-bearing plants as another possible dispersal mechanism

The egg stage is only 40 days for *Mogannia minuta* [12], whereas it is close to one year for other cicadas [6]. Female cicadas generally lay eggs in dead, decaying wood, but *M*. *minuta* deposits eggs on sugarcane leaves [6]. Such material (wood or leaves) may be rafted on the Kuroshio current (Fig 1), and eggs can be transported from a southerly island to a more northerly one.

We suggested that *Carabus blaptoides* beetles from central Kyushu were carried on driftwood by a major flood on 11–14 July 2012 to the ocean, then transported by rafting on the Kuroshio current to eventual landfall on Nii-jima islet, of the northern Izu islands [33]. The rafting took only ten days, and the beetle can apparently survive that duration of rafting.

For *Cryptotympana facialis*, rafting of eggs on/in driftwood on the Kuroshio current from Yaeyama especially to Tokara (Fig 1) at 0.58 Ma (Fig 6) may have been possible. Similar transport of *Meimuna boniensis* by rafting on the Kuroshio and then Ogasawara currents from Okinawa, through the Izu islands, to the Bonin islands (Figs 4 and 10) may have possible. *Meimuna kuroiwae* may have potentially rafted on the Kuroshio current from the Amami islands to Cape Sata (Figs 4 and 10) at 0.54 Ma (Fig 6).

There are several problems with rafting as a cicada dispersal mechanism. The first concerns the derivation of the driftwood from the original island, although the above are lowland and coastal species (Fig 1). Survival of eggs in seawater for at least ten days is also uncertain. A final problem is the vulnerability of hatched nymphs on material washed up on a beach. They may have to crawl a fairly long distance to reach the forested cover where they would burrow. Owing to these issues, we suspect that typhoon dispersal may be more likely than rafting dispersal for cicadas.

#### Artificial dispersal

For *Cryptotympana facialis*, human transplanting, of nymphs within the soils around roots or branches with eggs was proposed for the mechanism of their invasion into the Amami islands from the Okinawa islands [9, 10]. We revisit their proposal, because their paper was published in journal of limited circulation and in Japanese.

*Cryptotympana facialis* was not recorded in the Amami islands (Fig 1) from at least 1959 until 1990 [2, 3]. However, the first record of 1991 was from the Seisui park, Setouchi, southern Amami Oshima [34]. None of the recorded sightings in 1991 by Fukuda [3] was from northern Amami Oshima.

A modern ferry from Okinawa, Toku to Amami began service in 1972, and a ferry from Amami to Kikai-jima started service in 1974, whereas there has been no direct ferry from Okinawa to Kikai-jima (Fig 1). The development of these routes may have been related to the return of Okinawa from the USA in 1972, and the following economic development funded by the Japan government. On the Amami islands, ports, airports, roads, public facilities including urban parks were built, and extensive landscaping along shores and valleys was undertaken. Parks were planted with trees, and flowering trees such as *Erythrina variegata* were imported from Okinawa by the ferries connected with Amami. Note that domestic transportation of the flowing trees with soils around roots was allowed by the Plant Quarantine Station, Japan. *C*. *facialis* on the Amami islands was first recorded at or near newly constructed urban parks in low-lying areas, several years after Okinawa trees were planted. Because cicada nymphs are usually underground for several years, and eggs hatch the next year, so a lag time between the actual transplanting and adult emergence may have been several years. Table 2 summarized dates of the first collection of *C*. *facialis*, and the coincidence of the planting and the first adult emergence dates supports the hypothesis of the artificial dispersals from Okinawa at least for Amami Oshima.

**Table 2:** Summary of dates of first records of *Cryptotympana facialis* and transplantation dates for the Amami islands after references shown on this table. For land formation (human landscaping) dates, documentation by archival aerial photos offered by Geospatial Information Authority of Japan are referenced.

island	town	park	first record	land formation date	transplantion date from Okinawa	reference
Amami Oshima	Naze	Oshuku-central	1992 or 1994 July	1980–1982	1993 December- 1994 March	[9]Fukuda & Morikawa (1999)
		Oshuku-first	1992 or 1994 July	1980–1982	1991 January-March; 1991 August- 1992 March	[9]Fukuda & Morikawa (1999)
		Wauchi	1993	1989 December-1990 March	1989 December-1990 March	[36]Kanai (2008)
	Setouchi	Seisui	1991	<1984 (aerial photo)	1986 also from Kasari	[10]Fukuda et al. (2006)
Toku	Isen	Kinen	1997 (after 1997, 2003)	<1985 (aerial photo)	?	[9]Fukuda & Morikawa (1999)
	Amagi	Okamae	2005	1986	1981 (Hedono from Nakijin, Okinawa); 1985–1988	[35]Fukuda et al. (2009)
Kikai	Wan	Airport	2008	<2008 (aerial photo)	?	[37]Yoshiyuki & Matsuhira (2008)

As pointed out by [9, 35], the Amami population lacks the abdominal white band, as is the case for the Okinawa population. The present study showed that COI sequence of most of the Amami and Toku populations except for the Setouchi, a part of southern Amami population, is common to the sequence Okinawa population. These are concordant to the above observation summarized in Table 2 and their explanation.

For *Mogannia minuta*, that lays eggs on sugar cane, transplanting with nymphs and eggs from Miyako-jima or Iriomote-jima may be expected to result from modern shipping. However, sugar cane harvesting is mostly by cutting (rather than uprooting), and the import from another island is not necessary owing to self-sufficient crops. In addition, sugar cane and the root is lost after the harvest from the field, and nymphs usually cannot survive after the harvest. In contrast, the habitat for *Miscanthus sinensis* is adjacent to the sugar cane field, and we observed and collected *M*. *minuta* there. *M*. *minuta* is densely crowded on leaves, and the typhoon wind may be able to carry multiple cicadas from the original southern Iriomote-jima onto the new Yagachi-jima colony.

#### Alternative proposal for absence of *Cryptotympana facialis* on the Amami islands

In addition to long distance dispersal possibly by typhoon winds, we need to discuss the genetic homogeneity with a uniform COI sequence covering an extensive region that includes the Japanese, Osumi, and Tokara islands and encompassing more than 100 specimens, except for 4 specimens differing by one base substitution (Fig 1). After the dispersal to locations within the Japanese, Osumi, and Tokara islands at 0.58 Ma (Fig 6), *C*. *facialis* should have extensively radiated and expanded from original released positions. The genetic similarity may reflect bottleneck or founder effect, and the following range expansion.

The above consideration suggested that wide range of *Cryptotympana facialis* in the Japan, Osumi, and Tokara islands developed after 0.58 Ma. Accordingly, the Amami islands south of the Tokara islands may have originally lacked this species before 0.58 Ma.

**Fig 15:**
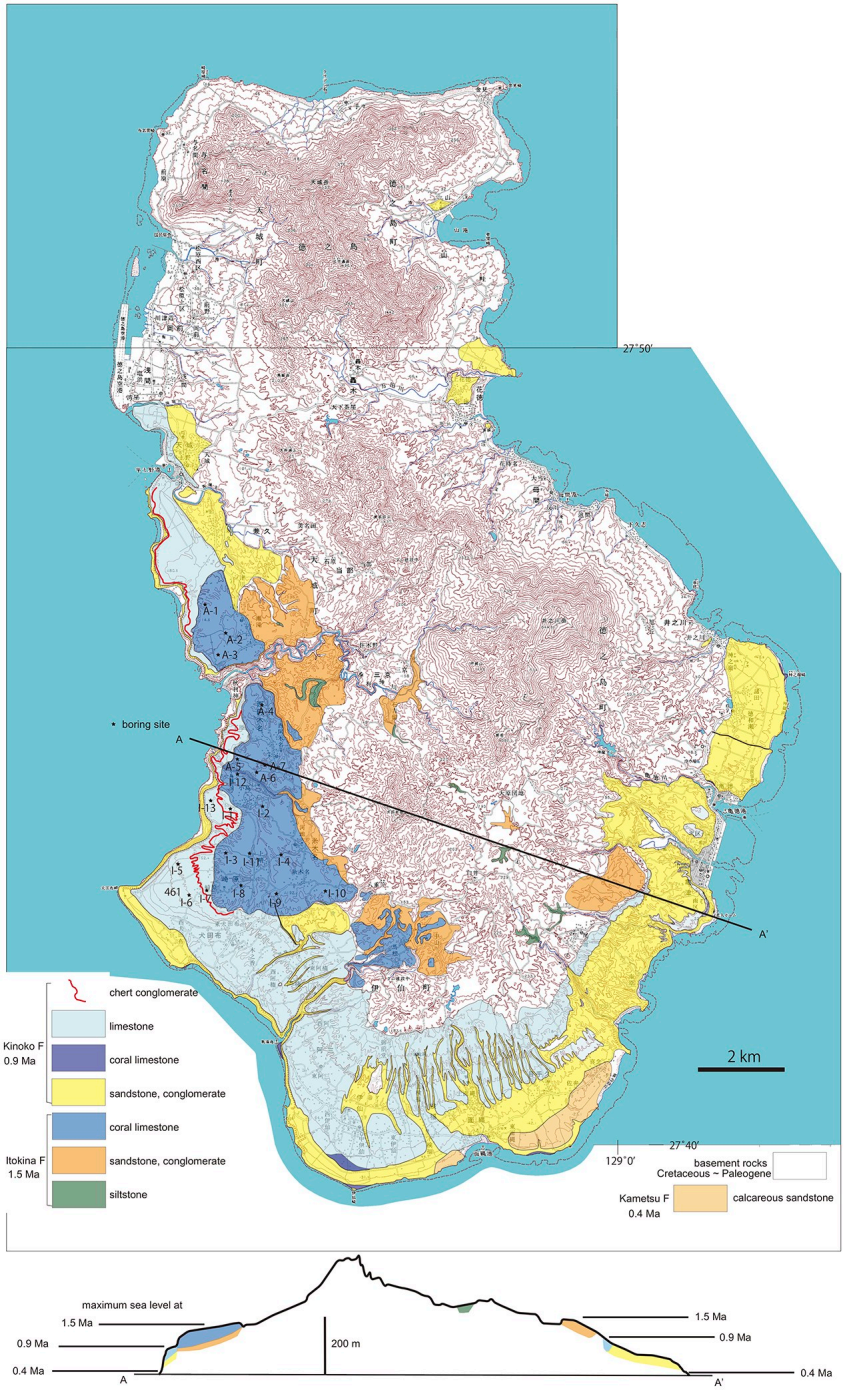
Geological map and cross section (below) of Tokuno-shima island. Three terraces consisting of the 1.5, 0.9 and 0.4 Ma limestone show three episodes of uplift or progressive uplift with erosion of wavecut benches during high sea level stands. Note the lack of coastal plains at 0.9 and 0.4 Ma, with development of 100-m-high sea cliffs instead. Background: Digital topographic maps of 1: 25,000 scale, the Geospatial Information Authority of Japan.

**Fig 16:**
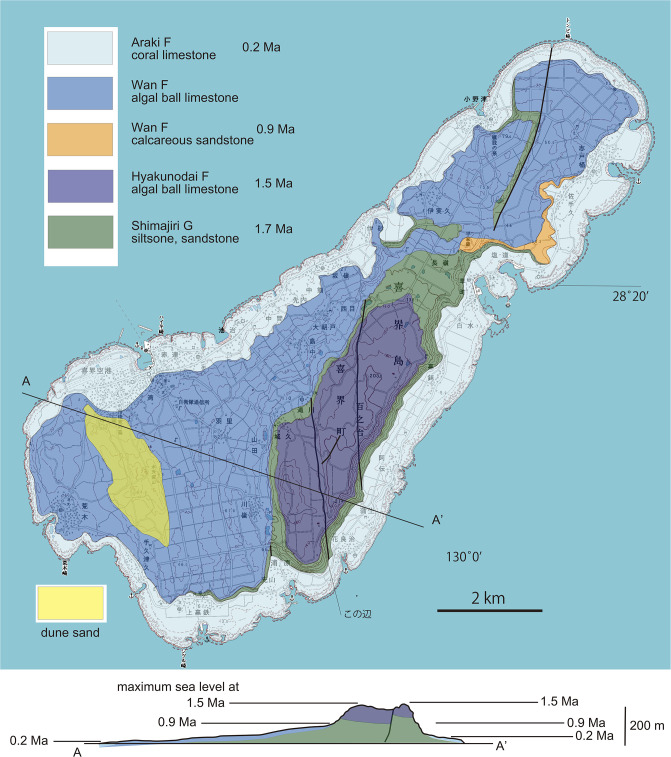
Geological map and cross section (below) of Kikai-jima island. Three terraces of the 1.5, 0.9 and 0.2 Ma limestone show three episodes of uplift or progressive uplift with erosion of wavecut benches during high sea level stands. Note the lack of a coastal plain at 0.9 Ma (100 m high sea cliff), but a relatively wide coastal plain at 0.2 Ma above a lower sea cliff. Background: Digital topographic maps of 1: 25,000 scale, the Geospatial Information Authority of Japan.

**Fig 17:**
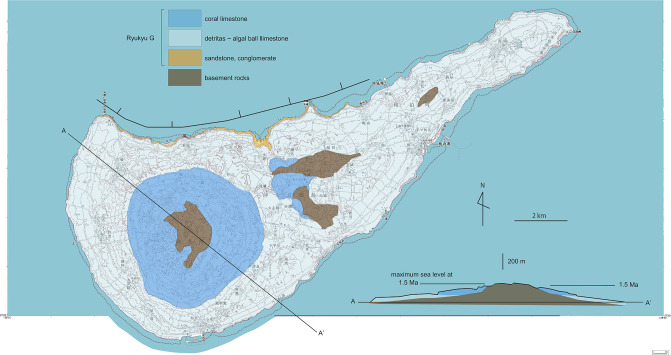
Geological map and cross section (below) of Okinoerabu-jima. A terrace developed on 1.5 Ma limestone. Uplifting was apparently slower compared to the examples of Toku-shima and Kikai-jima and has been progressed since 1.55 Ma. This history has resulted in a relatively wide coastal plain lacking high sea cliffs, except for fault scarp cliff at along the western part of northern margin. Miyako-jima and Ishigaki-jima have also only a single terrace in 1.5 Ma limestone, and northeastern end of Amami Oshima is also associated with 1.5 Ma limestone, whereas Okinawa-jima and Kume-jima have low elevatioon terraces developed with ca. 0.2 ~ 0.4 Ma (not dated) limestone. Background: Digital topographic maps of 1: 25,000 scale, the Geospatial Information Authority of Japan.

We propose, however, an alternate explanation for why *Cryptotympana facialis* was absent in the Amami islands prior to 1991. This species inhabits coastal lowlands less than 100 m in altitude in the Ryukyu islands including the post-1991 habitat on the Amami islands. The Toku-Kikai islands and Okinoerabu island have a distinct uplift history (Fig 1; [18] and modified). The Toku-Kikai islands have three marine terraces recording uplift events at 0.9, 0.4 or 0.2 Ma, and relatively recent (Figs 15 and 16) or alternatively reflecting relatively fast progressive uplift with erosion of wavecut benches (future terraces) during high stands of sea level. The uplift of these islands is an apparent consequence of the collision of the Amami and Daito plateaus with the west-dipping subduction zone that passes beneath the Toku-Kikai islands. On the Toku-Kikai islands this uplift history has resulted in a landscape lacking in lowland habitat that features seacliffs or terrace-riser cliffs (cliffs bounding marine terraces) instead (Figs 15 and 16). As a result, had there been an original *C*. *facialis* population, it may have been eliminated by tectonic (geologic) destruction of habitat on the Toku-Kikai islands. In contrast, Amami Oshima, except for its northeastern end with a terrace, has subsided so that upland areas rise from the coast instead of having the more gentle topography that typifies the ideal lowland habitat of *C*. *facialis*. Although Okinoerabu-jima has been uplifted, the landscape evolution is different than the Toku-Kikai islands that have concentric marine terraces and associated high cliffs. Okinoerabu-jima has a wide lowland region consisting by a single gentle terrace, and lacks sea cliffs except for the western part of the northern shore where there is a sea cliff that has resulted from an active fault scarp. The Okinoerabu geomorphic features have formed since the inception of uplift at 1.55 Ma and likely reflect a much lower rate of uplift compared to Toku-shima and Kikai-jima (Fig 17).

Accordingly, the lack of *C facialis* in the Amami islands prior to 1991 introduction may have been a consequence of the species not previously inhabiting the islands, or the species may have once populated parts of these islands but vanished when its habitat was destroyed by vertical crustal movements associated with the tectonics of this Amami region, especially Toku-Kikai.

## Conclusions

*Cryptotympana*, *Euterpnosia*, *Mogannia*, and *Meimuna* cicadas in the Ryukyu islands and the other parts of east Asia experienced the vicariant speciation due to the physical isolation of these islands from the Chinese continent since 1.55 Ma (Quaternary), and this tectonic and related evolutionary process continue in this region. Some cicadas were, however, transported distances of up to more than 1000 km in both recent (historic) and ancient (pre-historic) times. Winds associated with super typhoons that approached and passed through the Ryukyu islands may have been the key agent of the long-distance cicada dispersal. Because area of the present East China Sea was east Asian Chinese mainland before 1.55 Ma, typhoons would have lost energy upon landfall. In contrast, after the formation of the East China Sea by back-arc rifting (sea-floor spreading) starting at 1.55 Ma, typhoons would amplify or maintain their strength in this region.

## Supporting information

S1 Text (TXT)
